# Pleistocene Speciation in North American Lichenized Fungi and the Impact of Alternative Species Circumscriptions and Rates of Molecular Evolution on Divergence Estimates

**DOI:** 10.1371/journal.pone.0085240

**Published:** 2013-12-26

**Authors:** Steven D. Leavitt, H. Thorsten Lumbsch, Soili Stenroos, Larry L. St. Clair

**Affiliations:** 1 Committee on Evolutionary Biology, University of Chicago, Chicago, Illinois, United States of America; 2 Science & Education, The Field Museum, Chicago, Illinois, United States of America; 3 Department of Biology and M. L. Bean Life Science Museum, Brigham Young University, Provo, Utah, United States of America; 4 Botanical Museum, Finnish Museum of Natural History, University of Helsinki, Helsinki, Finland; University of Massachusetts, United States of America

## Abstract

Pleistocene climatic fluctuations influenced patterns of genetic variation and promoted speciation across a wide range of species groups. Lichens are commonly found in habitats that were directly impacted by glacial cycles; however, the role of Pleistocene climate in driving speciation in most lichen symbionts remains unclear. This uncertainty is due in part to limitations in our ability to accurately recognize independently evolving lichen-forming fungal lineages and a lack of relevant fossil calibrations. Using a coalescent-based species tree approach, we estimated divergence times for two sister clades in the genus *Xanthoparmelia* (Parmeliaceae) restricted to western North America. We assessed the influence of two different species circumscription scenarios and various locus-specific rates of molecular evolution on divergence estimates. Species circumscriptions were validated using the program BP&P. although speciation was generally supported in both scenarios, divergence times differed between traditional species circumscriptions and those based on genetic data, with more recent estimates resulting from the former. Similarly, rates of evolution for different loci resulted in variable divergence time estimates. However, our results unambiguously indicate that diversification in the sampled *Xanthoparmelia* clades occurred during the Pleistocene. Our study highlights the potential impact of ambiguous species circumscriptions and uncertain rates of molecular evolution on estimating divergence times within a multilocus species tree framework.

## Introduction

Improved species recognition has important implications for understanding diversity, ecological and biogeographical patterns, factors promoting diversification, and conservation [1-5]. While utilizing data from independent sources (ex. behavior, ecology, genetic, morphology, etc.) can facilitate the identification of species boundaries [2,6,7], integrating diverse data into phylogenetic and population genetic frameworks remains challenging [7]. However, modern molecular techniques allow researchers to effectively and quickly generate a large number of unlinked genetic markers from a large number of individuals (ex [8].). This information, coupled with the advent and maturation of algorithms for estimating species trees using multilocus sequence data, now provides unprecedented insights into species delimitation and the processes of speciation [1,9,10]. 

The recent union of coalescent theory with phylogenetics allows gene and species trees to be estimated simultaneously [9,11-13]. Multispecies coalescent-based species tree methods facilitate an effective approach for integrating multiple genetic loci from multiple individuals per species by modeling the coalescent gene histories embedded in a shared species tree [12,14,15]; ultimately, providing a substantial improvement over single gene trees or concatenated multilocus phylogenies [11,12,14-20]. Furthermore, coalescent-based species tree methods can accurately depict relationships even in cases where incomplete lineage sorting and gene tree heterogeneity (e.g. branch length and topology) obscures phylogenetic relationships among species [13,21,22]. In addition to improvements in estimating relationships in speciation histories, species tree methods also provide a more biologically realistic framework for temporal estimates of speciation events [23]. Recent modifications to the program BEAST [24] allow for joint estimation of divergence times and species trees [12]. Divergence dates estimated from concatenated gene trees can lead to overestimates of divergence times because gene divergence necessarily predates species divergence [23,25-27]. Furthermore, dating speciation events using priors on substitutions rates in a full probabilistic coalescent species-tree framework is especially relevant for studies investigating taxonomic groups with poor fossil records [23].

Most currently available species tree methods require that species/population assignments are made *a priori* [12,13,17,28]. However, correct assignments of samples to species may be difficult in a number of scenarios, including cryptic speciation [5,29], incongruence between morphology-based species and molecular data [30,31], or simply the fact that accurate specimen identification is a nontrivial task, particularly in complex groups with subtle or difficult to discern diagnostic characters. Inaccurate specimen identifications and equivocal, or contradictory, taxonomy may dramatically impact species tree and divergence time estimates where *a priori* species/population assignments are required. Objective assessments of alternative species delimitation hypotheses will likely result in improved perspectives on speciation histories and increased taxonomic stability [6]. Coalescent-based species delimitation methods provide a means for testing alternative hypotheses of evolutionary independence using multilocus genetic sequence data [6,32,33], and several approaches have recently been developed [13,28,32,34]. The program Bayesian Phylogenetics and Phylogeography (BP&P) delimits species using Bayesian model selection to calculate posterior probabilities of different species delimitation models using a reversible-jump Markov chain Monte Carlo (rjMCMC) [32]. This approach has been shown to outperform other coalescent-based species delimitation methods in a number of scenarios [33]. 

Lichens are obligate symbiotic associations consisting of a filamentous fungus and a photosynthetic partner (eukaryotic alga and/or cyanobacterium). Although the lichenized condition has been ecologically and evolutionarily successful [35-37], processes driving diversification in these symbiotic systems are poorly understood. Due to a poor fossil record and uncertainties in the interpretation of the few known lichen fossils [38-41], placement of diversification events within a temporal context remains challenging. 

Assessing diversification and biogeographical patterns in lichenized fungi is further confounded by uncertainties in species boundaries. In a number of cases, traditional phenotype-based species boundaries fail to accurately characterize diversity (reviewed in 42-44). For example, in some lichen-forming genera such as *Melanelixia* and *Melanohalea* (Parmeliaceae), diagnosable phenotypic differences appear to be absent even millions of years after the initial divergence, and morphologically similar species may not be each other’s closest relatives [45,46]. In contrast, other species of lichenized fungi have been shown to harbor a high degree of intraspecific morphological and chemical variation (ex [47-49].). 

The lichen-forming fungal genus *Xanthoparmelia* (Vain.) Hale (Parmeliaceae) provides an excellent study system with which to assess the impact of equivocal species delimitation scenarios and locus-specific evolutionary rates for estimating divergence times in a coalescent-based species tree framework. *Xanthoparmelia* is among the most speciose genera of lichen-forming fungi, including ca. 820 species [50]. A number of studies have provided novel insights into species relationships as well as biogeography and character evolution within the genus [48,51-57]. Although fossil evidence for *Xanthoparmelia* has not yet been found, taxon-specific (either family- or genus-level) substitution rates have been estimated for four loci – nuclear ribosomal ITS, nuclear ribosomal LSU, RPB1, and mitochondrial SSU [25,45,58]. In Parmeliaceae, rate- and fossil-calibrated phylogenies have resulted in similar estimates of divergence times for a number of deeper lineages within the family [25,45,56,58,59], supporting the use of rate-calibrated divergence estimates. 

Large regions in western North America were subjected to global cooling, aridification, and major glaciation events during the Pleistocene [60-62]. These climatic shifts had a substantial impact on both vascular plant and lichen communities [25,63-67]. Additionally, Pleistocene climatic fluctuations appear to have played a major role in speciation for at least some lichen-forming fungi in western North America [64]. In contrast to Pleistocene-dominated speciation, a number of recent studies suggest that the Neogene was a major period of diversification for extant lineages in the most speciose lichen-forming fungal family, Parmeliaceae [25,45,56,58,59,68]. Based on limited sampling, species-level diversification coinciding with Pleistocene glacial cycles appears to be restricted to a limited number of clades within some parmelioid genera [25,68]. 

To better understand the impact of Pleistocene climate change on speciation in lichenized fungi, we assessed the timing of diversification in two *Xanthoparmelia* clades that are commonly distributed across a wide range of montane habitats, including, shrub-steppe, subalpine, and alpine communities in western North America (clades ‘D’ and ‘E’ in [48,52]). The speciation histories of these *Xanthoparmelia* clades remain unsettled due to ambiguities in species boundaries and incomplete lineage sorting [48,52]. Both clades include phenotypically polymorphic forms, representing a total of 15 traditionally circumscribed species [48,52]. In contrast to the 15 phenotype-based species recovered in these two clades, multilocus sequence data support a total of four polymorphic species-level genetic clusters. The majority of the traditionally circumscribed species recovered in clades ‘D’ and ‘E’ are also found in other distinct lineages [48]. However, formal taxonomic changes have not yet been made. 

The general lineage concept [69-71] provides a framework for species delimitation using a variety of operational criteria, data sets, and analytical methods to circumscribe segments of separately evolving metapopulation lineages. In this study, we estimated speciation probabilities for both traditional, phenotype-based species and molecular-based genetic groups using the coalescent-based species delimitation method BP&P [32]. We then compared divergence times using rate calibrated species trees, based on the two different species delimitation hypotheses, and evaluate the impact of substitution rates for different loci. The impact of equivocal species circumscriptions on divergence times has not been evaluated within a coalescent framework, and our study provides a valuable perspective on the impact of species delimitations and rates of molecular evolution for estimating divergence times within a species tree framework. In addition, our results offer insights into the speciation history of *Xanthoparmelia* species in western North America. 

## Materials and Methods

### Sampling

We sampled a total of 180 individuals (Table S1) from two previously recognized sister clades, ‘clade D’ and ‘clade E’, which appear to be restricted to western North America [48,52]. Specimens were obtained across a variety of habitats using both opportunistic field sampling and herbarium collections. Lichens collections were made on public lands managed by the Bureau of Land Management and USDA Forest Service, and sampling permits were not required. Traditional specimen identifications were based on vegetative morphological characters and diagnostic medullary extrolites identified using thin layer chromatography (TLC; [72,73]), following established protocol [74,75]. ‘Clade D’ was shown to be dominated by saxicolous, isidiate morphs (*X*. aff. *dierythra*, *X*. aff. *mexicana*, *X*. aff. *plittii*, and *X*. aff. *subplittii*), but also included a limited number of non-isidiate specimens (*X*. aff. *lineola*) [48]. ‘Clade E’ included eight non-isidiate species (*X*. aff. *californica* [saxicolous], *X*. aff. *chlorochroa* [vagrant], *X*. aff*. coloradoënsis* [saxicolous], *X*. aff. *cumberlandia* [saxicolous], *X. lipochlorochroa* Hale & Elix [vagrant], *X*. aff. *neochlorochroa* [vagrant], *X*. aff. *norchlorochroa* [vagrant], *X*. aff. *vagans* [vagrant], and *X. wyomingica* (Gyelnik) Hale [semi-attached]), distributed across three distinct genetic clusters (‘E1’, ‘E2’, and ‘E3’; [52]). *Xanthoparmelia lipochlorochroa* and *X. wyomingica* were represented by collections made from the type localities. None of the sampled species are protected by United States law.

### DNA extraction, amplification and sequencing

DNA was extracted from all individuals in connection with previous studies [48,52], and we generated DNA sequence data for a total of nine markers. Four nuclear ribosomal loci were sampled, including the large-subunit (nrLSU, *c.* 844 bp), the internal transcribed spacer region (ITS, *c.* 548 bp), a group I intron (*c.* 398 bp), and the intergenic spacer region (IGS, *c.* 380 bp). In addition, we sampled five protein-coding loci, β-tubulin (787 bp), glyceraldehyde 3-phosphate dehydrogenase (GAPDH, 598 bp), MCM7 (541 bp), RPB1 (807 bp), and RPB2 (823 bp). Amplification of the nrLSU, IGS, ITS, group I intron, β-tubulin, and MCM7 was described previously [52]. For this study, we amplified three additional protein-coding markers, the GAPDH, RPB1, and RPB2. The GAPDH fragment was amplified using the following primers Gpd1-LM with Gpd2-LM [76]; the RPB1 fragment was amplified using gRPB1-A [77] and fRPB1-C [78]; and primers fRPB2-5f with fRPB2-7cr were used to amplify the RPB2 fragment [79]. PCR amplifications were performed using Ready-To-Go PCR Beads (GE Healthcare, Pittsburgh, PA, USA), and cycling parameters followed a 55–50 °C touchdown reaction [80]. PCR products were visualized on 1% agarose gel and cleaned using ExoSAP-IT (USB, Cleveland, OH, USA). We sequenced complementary strands using the same primers used for amplifications, and sequencing reactions were performed using BigDye 3.1 (Applied Biosystems, Foster City, CA, USA). Products were run on an ABI 3730 automated sequencer (Applied Biosystems) at the Pritzker Laboratory for Molecular Systematics and Evolution at the Field Museum, Chicago, IL, USA.

### Alignment

We assembled and edited new sequences using Sequencher 4.10 (Gene Codes Corporation, Ann Arbor, MI). Sequences were aligned using the program MAFFT 6 [81,82]. We implemented the G-INS-i alignment algorithm and ‘1PAM / K=2’ scoring matrix, with an offset value of 0.9, and the remaining parameters were set to default values for the protein-coding (β-tubulin, GAPDH, MCM7, RPB1 and RPB2) and nrLSU markers. For the ribosomal ITS, IGS, and group I intron loci, we used the same parameters, with the exception of an offset value set to 0.1 rather than 0.9.

### Population structure analyses

Previous studies have suggested that traditional morphology/chemistry-based taxonomy may misrepresent *Xanthoparmelia* diversity in western North America [48,51]. Therefore, we used multilocus Bayesian population assignment tests implemented in the programs STRUCTURE 2.3.2 [83,84] and BAPS 5 [85] to identify genetic groups as an alternative to traditional phenotype-based species. Varying approaches have been implement to convert DNA sequence data to allelic data for Bayesian clustering (see 86). STRUCTURE is expected to perform well when there is sufficient independence across regions such that linkage disequilibrium within regions does not dominate the data (STRUCTURE manual), but can also be effective using multilocus sequence data and treating all SNPs as independent loci regardless of physical linkage within each locus (eg. 84-87). In order to assess the impact of using linked genotyped SNP data from ribosomal markers, we compared three genotyped data sets created from the aligned sequence data. The first comprised SNP data exclusively from ribosomal tandem repeat (nrLSU, ITS, IGS, group I intron); the second comprised SNPs from putatively unlinked protein-coding loci (β-tubulin, GAPDH, MCM7, RPB1, and RPB2); and the final genotype dataset comprised SNPs from all sampled loci [87-89]. STRUCUTRE analyses were run for clades ‘D’ and ‘E’ separately. In the STRUCTURE analyses, we ran 10 replicate runs for each *K* value, from 1–6, using 50,000 MCMC generations for the burn-in period and an additional 50,000 generations to estimate the posterior distribution. We used an admixture model that incorporated the possibility for individuals to have mixed cluster ancestry, including an ‘F model’ to account for correlated allele frequencies among populations resulting from migration or shared ancestry [84]. We used the program STRUCTURE HARVESTER [90] to process the results from the 10 replicate runs for each *K* value. The Δ*K* procedure outlined by [91] was then used to estimate the most likely number of clusters within the sample. However, we also considered other values of *K* that seemed biologically sensible (see STRUCTURE manual). We classified individuals with posterior probabilities < 0.70 to any cluster as “admixed”. We also used Bayesian clustering in BAPS [85] to examine the number of groups. BAPS uses a stochastic optimization procedure to identify the number of groups and is capable of handling sequence data by utilizing a linkage model [85]. In contrast to STRUCTURE, BAPS includes *K* as a parameter to be estimated, and the best partition of the data into *K* clusters is identified as the one with the highest marginal log likelihood. We selected the clustering with linked loci analysis and replicated the analysis ten times to ensure the stability of our results. Additionally, we ran BAPS with the number of clusters fixed at *K* = 5 (see STRUCTURE results below) and then replicated multiple times to ensure stability. 

### Phylogenetic analyses

We used the program RAxML 7.2.8 [92,93] to reconstruct maximum likelihood (ML) gene trees from each of the individual gene alignments. A search combining 200 separate ML searches was conducted, implementing the ‘GTRGAMMA’ model, and 1000 pseudoreplicates to evaluate bootstrap support for each node. Relationships were also estimated from the concatenated nine-locus data matrix using a total-evidence approach [94]. We conducted an ML analysis of the concatenated data set using locus-specific model partitions in RAxML, and all loci were treated as separate partitions. Search parameters and assessment of nodal support were performed as described above. In order to assess differences between ribosomal and protein-coding loci for tree reconstruction, we conducted separate ML analyses of the complete ribosomal dataset (ITS, nrLSU, IGS, and group I intron) and the 5-gene protein-coding dataset (β-tubulin, GAPDH, MCM7, RPB1, and RPB2). In both cases we used locus-specific model partitions, and all loci were treated as separate partitions. Search parameters and assessment of nodal support were performed as described above.

### Species trees and divergence dating

Estimating a species tree using concatenated multilocus sequence data may be misleading under certain divergence scenarios [19,95,96]. In addition, divergence times are consistently overestimated in gene tree approaches, including concatenated loci, due to the fact that the time of gene divergence necessarily predates speciation events [23,26]. Therefore, we used the coalescent-based hierarchical Bayesian model *BEAST implemented in BEAST 1.7.4 to estimate species trees and divergence times [12]. *BEAST incorporates the coalescent process and the uncertainty associated with gene trees and nucleotide substitution model parameters and estimates the species tree directly from the sequence data [12]. Species assignments are required *a priori* for *BEAST analyses, and assignment errors can mislead species tree inference and result in strong support for an incorrect species tree [96]. Therefore, we explored two different scenarios for assigning individuals to a ‘species’ group. First, we chose to explore the relationships among all potential lineages inferred using molecular sequence data. We expected strong nodal support for well-differentiated lineages in concatenated gene tree and/or coalescent-based species tree analyses, in addition to high speciation probabilities separating distinct species-level lineages [32]. We used results from the STRUCTURE and ML analyses to assigned individuals to one of seven populations. For ‘clade E’, we treated each of the genetic clusters from the *K* = 5 model as distinct species because the STRUCTURE analyses indicate a general pattern of a plateau near *K* = 5 (see Results). Individuals with inferred admixed ancestry were excluded. Although the STRUCTURE analysis did not indicate distinct populations within ‘clade D’, the ML analysis of the concatenated data matrix recovered two well-supported clades (see Results), and previous studies have shown that clustering algorithms may fail to recover genetically distinct groups represented by unbalanced sample sizes [97,98]. Therefore, individuals from ‘clade D’ were assigned to two separate groups based on the results of the ML analysis. In the second scenario, we assigned all individuals to a species group based on traditional morphological characters. We included a total of 11 species, *X*. aff. *chlorochroa* (54 specimens), *X*. aff. *coloradoënsis* (34), X. aff. *cumberlandia* (43), X. aff. *lineola* (8), X*. lipochlorochroa* (3), X. aff. *mexicana* (17), X. aff. *neochlorochroa* (4), X. aff. *norchlorochroa* (2), X. aff. *plittii* (6), X. aff. *vagans* (4), and *X. wyomingica* (2). Based on previous results [48] and subsequent morphological re-examinations, specimens representing *X. neowyomingica* were combined with *X. cumberlandia*; and *X. subplittii* was merged with X. *plittii*. *Xanthoparmelia californica* and *X. dierythra* were each represented by a single specimen and therefore excluded from these analyses due to small sample sizes (see 21,99,100). 

We estimated divergence using *BEAST because species tree methods provide a more biologically realistic framework for dating divergence events by directly modelling genetic divergence that pre-dates speciation [23]. In the absence of relevant fossil evidence, we assessed four different scenarios using molecular locus-specific rates of evolution for Parmeliaceae. First, we used 2.43 × 10^−9^ substitution/site/year (s/s/y) for the ITS marker, recently reported for the parmelioid lichen-forming genus *Melanelixia* [45]. This rate is similar to other estimates of ITS substitution rates for lichen-forming mycobionts (2.38 × 10^−9^ s/s/y *Oropogon*, Parmeliaceae, Lecanorales [68]; and a non-lichenized fungus (2.52 × 10^−9^ s/s/y, Erysiphales; [101]. In the second scenario, we used a rate estimated for Parmeliaceae (*Protoparmelia* excluded) for the nrLSU (0.70 × 10^−9^ s/s/y, [58]. In the third scenario we used substitution rates for both the ITS (2.38 × 10^−9^ s/s/y) and nrLSU (0.70 × 10^−9^ s/s/y) markers. In the final scenario we used the RPB1 substitution rate (1.51 × 10^−9^ s/s/y) estimated for *Xanthoparmelia* [58]. In all cases, substitution rates for other loci were co-estimated along the run under a uniform prior (0 – 50), relative to the specified molecular evolution rates. Locus-specific models of evolution were selected using jModeltest 2 [102], and ultrametric trees were constructed using the most similar substitution models available in BEAST. Divergence times were estimated using an uncorrelated relaxed lognormal molecular clock used for all loci [103]. We also implemented a Yule process and gamma-distributed population sizes for the species tree prior and a piecewise linear population size model with a constant root. Default values were used for the remaining priors. For each scenario, we ran two independent Markov chain Monte Carlo (MCMC) analyses for a total of 200 million generations, sampling every 2000 steps. The first 25% of sampled trees were excluded as burn-in. We assessed convergence by examining the likelihood plots through time using Tracer 1.5 [104] and compared summarized tree topologies from separate runs. The posterior probabilities of nodes were computed from the sampled trees (excluding burn-in samples) using TreeAnnotator 1.7.4 [104].

### Speciation probabilities

We explored the validity of the two species delimitation scenarios. The first based on the seven groups inferred using molecular sequence data and the second treating traditionally circumscribed species as distinct species. The marginal posterior probability of speciation for each scenario was estimated using the program BP&P 2.1 [16,32]. This method accommodates the species phylogeny as well as lineage sorting due to ancestral polymorphisms. BP&P has recently been shown to outperform other coalescent-based species delimitation methods in cases of recent speciation events, with robust performance using a modest number of genetic markers [33]. We used the species trees inferred from the *BEAST analyses of the traditional species and genetic clusters as the guide trees and clades ‘D’ and ‘E’ were analyzed separately. In cases where relationships were not strongly supported in the coalescent-based species tree, we used different guide trees representing alternative topologies and assessed speciation probabilities among the topologies. The prior distributions on ancestral population size (θ) and root age (τ_0_) were assigned gamma distributions of G(1,100) and G(2, 2000), respectively, assuming intermediate ancestral population sizes and relatively shallow divergences among species [5,64]. We used algorithm 0 and each species delimitation model was assigned equal prior probability. Each reversible-jump Monte Carlo (rjMCMC) analysis was run 500,000 generations with a burn-in of 50,000. Each analysis was run at least twice to confirm consistency between runs. 

## Results

### Molecular data

The concatenated nine-gene data matrix consisted of 5726 aligned nucleotide positions (Table 1; TreeBase # 14929). Overall, 601 sites were variable, 273 in the ribosomal markers and 328 in the five protein-coding loci (Table 1).

**Table 1:** Genetic variability of sampled markers used in this study, including the total number of samples, *N* (number of haplotypes); aligned length; variable and parsimony-informative (PI) sites for each sampled locus; and model of evolution selected using the program jModeltest.

Locus	*N* (haplotypes)	Aligned bp	No. variable sites (PI sites)	Model
ITS	179 (76)	548	96 (61)	TrNef+G
nrLSU	176 (43)	844	42 (19)	TrN+I+G
IGS	164 (61)	380	68 (34)	K80+G
Intron	157 (61)	398	67 (41)	K80+G
β-tubulin	168 (42)	787	67 (33)	TrNef+G
GAPDH	155 (55)	598	56 (44)	SYM+I+G
MCM7	163 (74)	541	80 (45)	K80+I+G
RPB1	178 (35)	807	45 (30)	TrNef +I
RPB2	178 (77)	823	80 (59)	TPM1+I+G
All ribosomal loci	180 (125)	2170	273 (155)	NA
All protein-coding loci	180 (154)	3556	328 (211)	NA
All loci	180 (167)	5726	601 (366)	NA

### Bayesian clustering

The STRUCTURE analyses revealed little evidence in support of population substructure within ‘clade D’. Population clusters inferred for K > 1 did not correspond to morphological/chemical groups or geographic regions and tended to include a high number of individuals with inferred admixed ancestry (see Figure S1). The Δ*K* method indicated that a *K* = 2 model best fits the data; however, the same method does not identify the best *K* if *K* = 1. Furthermore, clustering using BAPS also yielded a single population cluster for ‘clade D’ (see Figure S1). 

**Figure 1:**
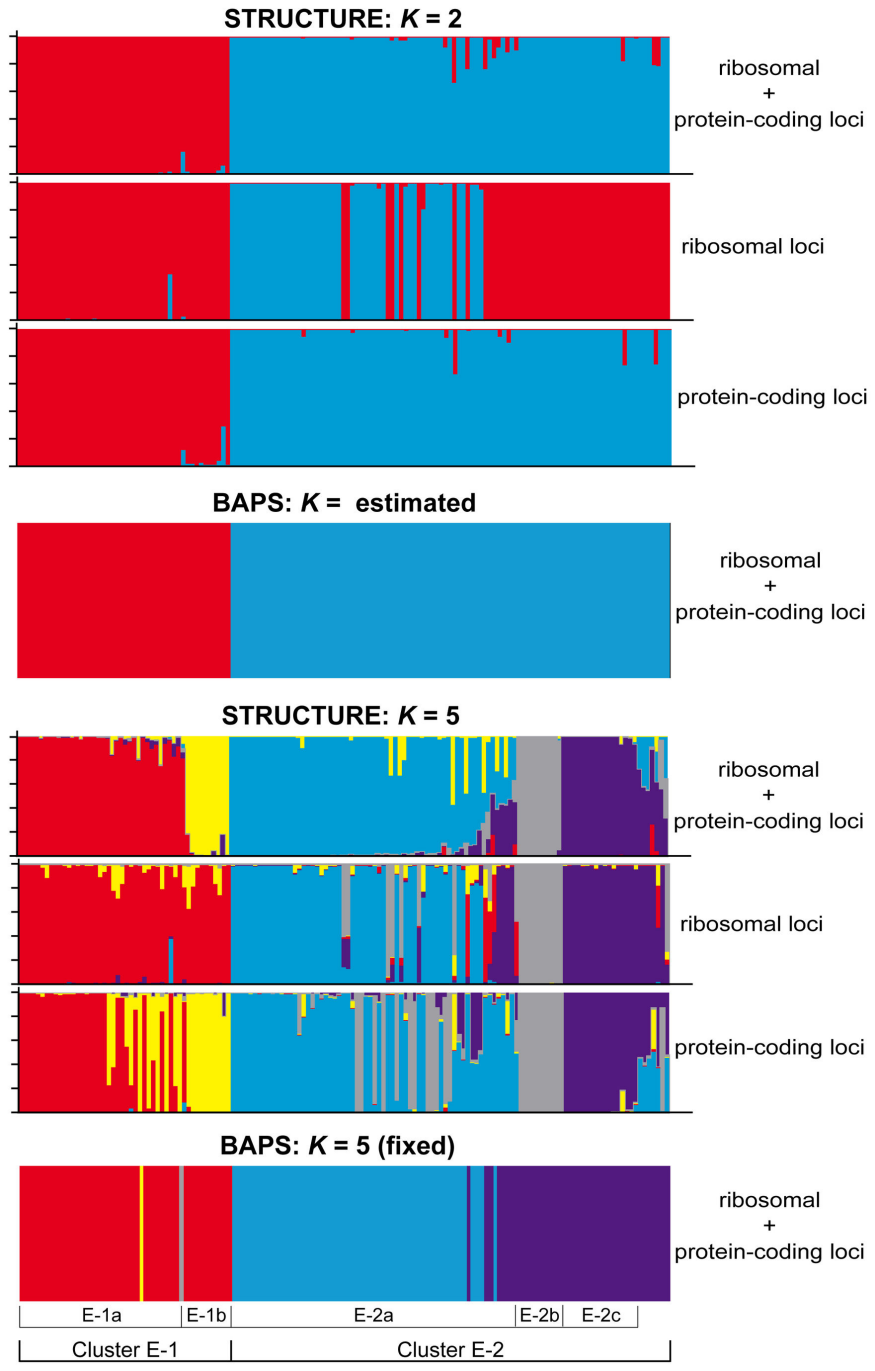
Population assignments to genetic clusters in *Xanthoparmelia* ‘clade E’. Population membership was inferred using the Program BAPS and STRUCTURE using SNP data from nine sampled loci. (nrLSU, IGS, ITS, group I intron, β-tubulin, GAPDH, MCM7, RPB1, and RPB2), ribosomal loci only (nrLSU, IGS, ITS, group I intron) and protein-coding loci only (β-tubulin, GAPDH, MCM7, RPB1, and RPB2). STRUCUTRE analyses include estimates for *K* = 2 and *K* = 5 models; and the BAPS analyses included the estimated number of clusters (*K* = 2) and the number of clusters fixed at *K* = 5. Each accession is shown by a thin vertical line that is partitioned into colored segments representing the proportion of each individual’s genome assigned to a genetic cluster.

For ‘clade E’, a comparison of the three genotyped data sets – SNPs from the ribosomal tandem repeat, SNPs from the protein-coding markers, and SNPs from all sampled loci – are shown in Figure 1. Both BAPS and the Δ*K* method, based on results from the STRUCTURE analysis, indicated that a *K* = 2 model best fits the data (see Fig. S2). The median ML values of the Bayesian clustering analyses based on the dataset comprised of SNPs from all sampled loci with estimates of *K* from 1–6 are reported in Figure S2. These analyses indicate a general pattern of a plateau near *K* = 5, and we chose also to examine the phenotypic expressions and geographic distributions of population clusters within the *K* = 5 model because individual genetic clusters were generally associated with distinct phenotypic or geographic patterns. Exploratory analyses of *K* > 5 revealed a larger proportion of individuals with admixed ancestry, relative to smaller *K* values, and did not seem biologically sensible (data not shown). The distribution of individual population cluster membership assignments for both the *K* = 2 and *K* = 5 models are shown in Figure 1. In the *K* = 5 model, all individuals assigned to cluster ‘X-1A’ produced stictic acid (*X*. aff. *cumberlandia*). In contrast, individuals assigned to cluster ‘X-1B’ produced salazinic acid (*X*. aff. *coloradoënsis*). The majority of individuals assigned membership in clusters ‘X-2A’, ‘X-2B’, and ‘X-2C’ also produced salazinic acid. However, a small proportion of individuals in cluster ‘X-2A’ produced stictic acid rather than salazinic acid, including both saxicolous (*X*. aff. *cumberlandia*) and vagrant morphs (*X*. aff. *vagans*) (Table 2). Cluster ‘X-2B’ was comprised exclusively of vagrant morphs collected from southwestern Wyoming and western Montana, and included vagrant species X. aff. *chlorochroa* (salazinic acid chemosyndrome), *X. lipochlorochroa* (fatty acid chemosyndrome) and X. aff. *neochlorochroa* (norstictic acid chemosyndrome). Cluster ‘X-2C’ was also comprised exclusively of vagrant morphs, *X*. aff. *chlorochroa*, although these originated from southern Utah and a single site in Colorado, USA. Approximately 20% of the individuals assigned to cluster ‘E-2’ in the *K* = 2 model, were recovered with admixed ancestry in the *K* = 5 model. 

**Table 2:** Estimated substitution rates for western North American *Xanthoparmelia* clades ‘D’ and ‘E’ from the *BEAST analysis based on population clusters inferred from SNP data from a nine-locus data set.

	nrLSU	ITS	RPB1	ITS/LSU
IGS	3.38 (HPD = 1.92 - 5.02)	1.95 (HPD = 1.27 - 2.68)	7.14 (HPD = 4.06 - 10.54)	2.10 (HPD = 1.43 - 2.83)
Intron	3.93 (HPD = 2.18 - 5.86)	2.28 (HPD = 1.45 - 3.16)	8.29 (HPD = 4.73 - 12.39)	2.44 (HPD = 1.61 - 3.31)
nrLSU	0.70 (fixed 0.70)	0.57 (HPD = 0.34 - 0.83)	2.07 (HPD = 1.09 - 3.18)	0.69 (fixed 0.70)
ITS	4.86 (HPD = 2.82 - 7.13)	2.42 (fixed = 2.43)	10.27 (HPD = 6.00 - 15.10)	2.43 (fixed 2.43)
β -tubulin	1.66 (HPD = 0.94 - 2.48)	0.96 (HPD = 0.62 - 1.33)	3.50 (HPD = 1.99 - 5.18)	1.03 (HPD = 0.69 - 1.40)
GAPDH	2.54 (HPD = 1.38 - 3.86)	1.48 (HPD = 0.92 - 2.13)	5.43 (HPD = 2.97 - 8.26)	1.60 (HPD = 1.02 - 2.24)
MCM7	4.61 (HPD = 2.55 - 7.02)	2.72 (HPD = 1.69 - 3.85)	9.87 (HPD = 5.54 - 14.73)	2.81 (HPD = 1.84 - 3.88)
RPB1	1.00 (HPD = 0.54 - 1.53)	0.58 (HPD = 0.35 - 0.83)	1.56 (fixed = 1.51)	0.62 (HPD = 0.39 - 0.87)
RPB2	2.45 (HPD = 1.38 - 3.65)	1.42 (HPD = 0.90 - 1.96)	5.17 (HPD = 2.97 - 7.68)	1.52 (HPD = 1.02 - 2.06)

Rates were estimated under an uncorrelated lognormal relaxed molecular clock using fixed substitution rates for four different scenarios (fixed rates shown in parentheses): nrLSU, ITS, RPB1, and ITS/nrLSU markers. Units: substitution/site/10^9^ years.

### ML topologies

**Figure 2:**
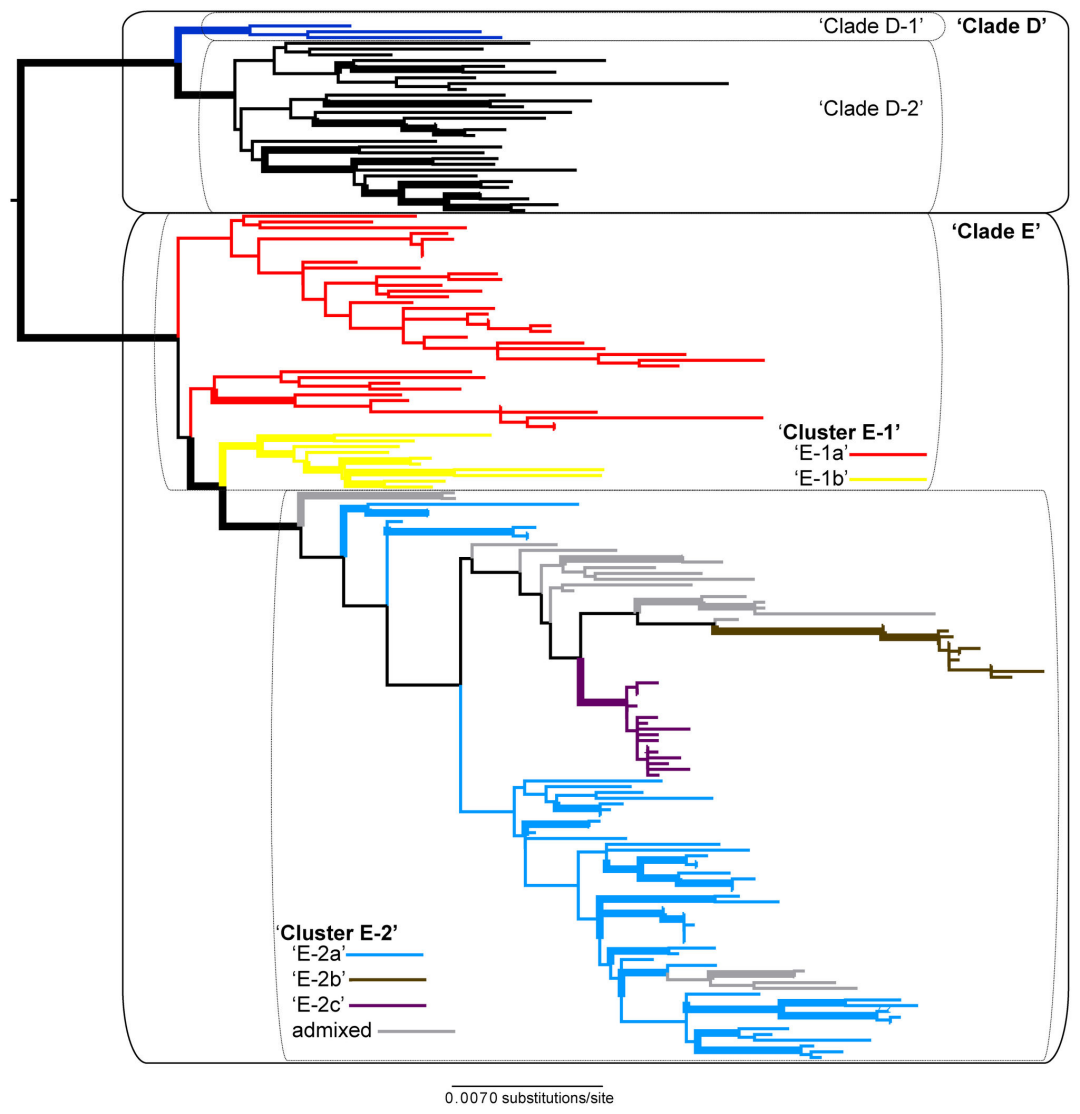
Maximum likelihood topology of *Xanthoparmelia* clades ‘D’ and ‘E’. Phylogeny estimated from ribosomal (nrLSU, IGS, ITS, and a group I intron) and protein-coding (β-tubulin, GAPDH, MCM7, RPB1, and RPB2) markers representing *Xanthoparmelia* clades ‘D’ and 'E’. Thickened branches indicate ML bootstrap values ≥ 70%, and colored branches correspond to population genetic clusters from the *K* =5 model in the STRUCTURE analyses (see Figure 1).

Neither traditional, phenotype-based species nor genetic population clusters were recovered as monophyletic in any of the individual gene topologies (Figures S3 A–I). The ML phylogeny estimated from the concatenated 9-locus phylogeny is shown in Figure 2 (see Figure S4 for complete labels). The 5-gene protein-coding phylogeny was more similar to the total evidence phylogeny than the 4-gene ribosomal phylogeny (see Figures S4, S5 & S6). The total evidence phylogeny revealed two well-supported lineages within ‘clade D’ (Figure 2). Three non-isidiate individuals producing salazinic acid were recovered within clade ‘D1-A’, while clade ‘D1-B’ was dominated chemically by polymorphic isidiate specimens (Figure S5). Two of the five population clusters from the *K* = 5 model, ‘E-1a’ and ‘E-2b’, were not recovered as monophyletic in the 9-gene phylogeny (Figure 2).

### Coalescent-based species trees and divergence time estimates

**Figure 3:**
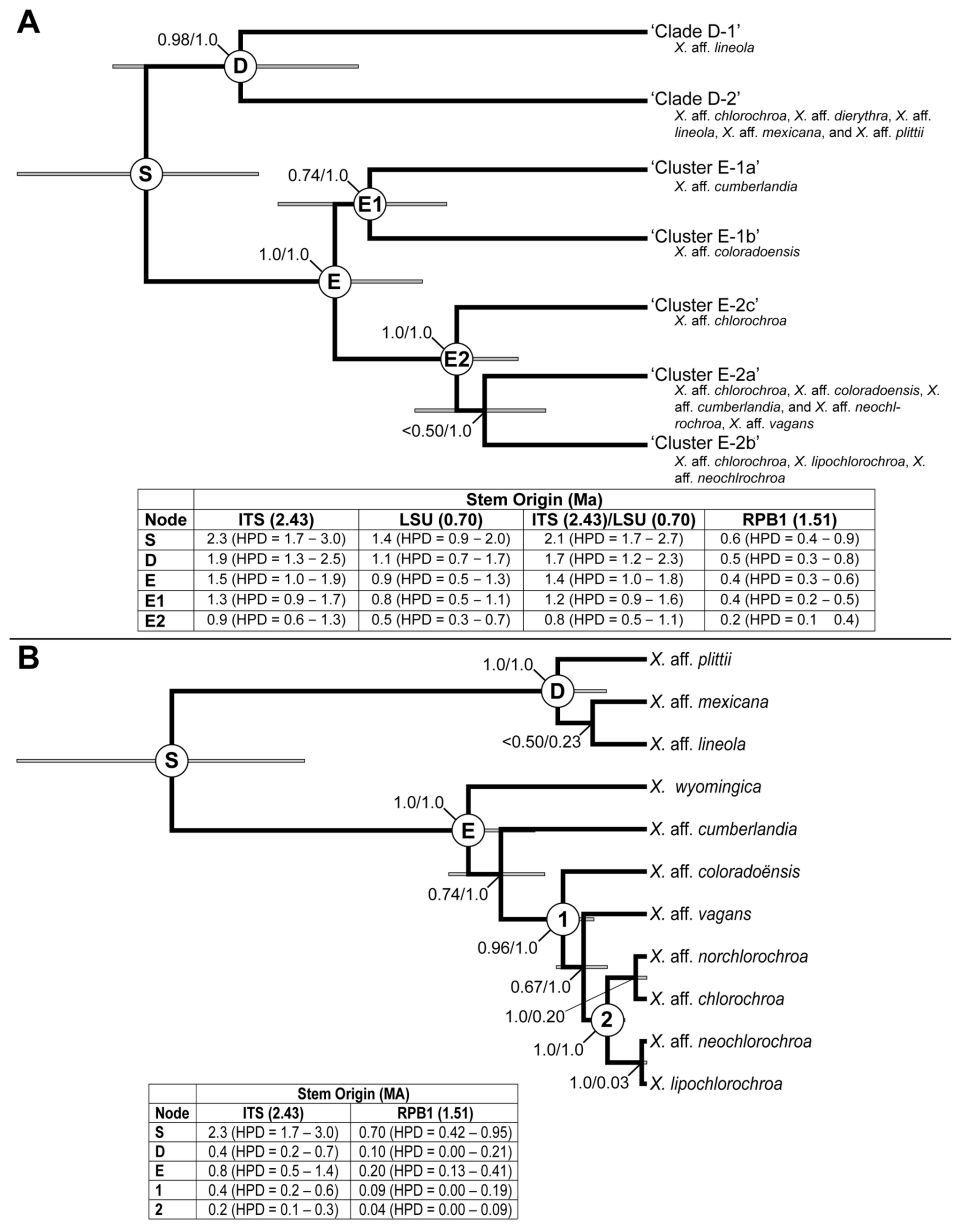
Time-calibrated species trees for *Xanthoparmelia* clades ‘D’ and ‘E’. Divergence estimates were based on (A) genetic population clusters and (B) traditional, morphology-based species circumscriptions. Posterior probabilities at nodes indicate support from the *BEAST analyses and speciation probabilities. Inset tables include divergence times for specified nodes, including the highest posterior density interval (HPD), using fixed substitution rates in four different scenarios: dates estimated using the ITS, nrLSU, RPB1 loci, independently; and the forth using rates from both the ITS nrLSU loci (Units: substitution/site/10^9^ years).

In the *BEAST analyses ESS values were > 150 for all parameters and the vast majority were well above 200. Topologies and divergence estimates were congruent across independent runs. A maximum clade credibility chronogram from the multi-locus species tree analyses based on the seven-species model inferred from the STRUCTURE and ML analyses is shown in Figure 3A. Similar to the ML analysis of the 9-locus data matrix, the *BEAST analysis supported a split between the two lineages within ‘clade D’. While within ‘clade E’, a sister relationship was recovered for clusters ‘E-1a’ and ‘E-1b’ with weak statistical support (posterior probability [PP] = 0.68). Clusters ‘E-2a’, E-2b’, and ‘E-2c’ were recovered within a well-supported (PP = 1.0) monophyletic lineage, corresponding to ‘cluster 2’ from the *K* = 2 STRUCTURE analysis. However, relationships among clusters within this clade were weakly supported (PP < 0.50). 

A maximum clade credibility chronogram from the multi-locus species tree analyses based on the traditional, phenotype-based species circumscriptions is shown in Figure 3B. Relationships among the three species within ‘clade D’ were unresolved (PP < 0.50). In clade ‘E’ a well-supported lineage (PP = 0.95) was recovered and included X. aff. *chlorochroa*, *X*. aff. *coloradensi*s, *X. lipochlorochroa*, *X*. aff. *neochlorochroa*, and X. aff. *norchlorochroa*. This clade generally corresponds to clusters ‘E-2a’, ‘E-2b’, and ‘E-2c’ from the *K* = 5 STRUCTURE analysis. However, relationships between this clade and *X*. aff. *cumberlandia* and *X*. aff. *wyomingica* were unresolved (Figure 3B). The vagrant species X. aff. *chlorochroa*, *X. lipochlorochroa*, *X*. aff. *neochlorochroa*, and X. aff. *norchlorochroa* were recovered as a well-supported (PP = 1.0) monophyletic clade, sister to X. aff. *vagans* (Figure 3B) with weak statistical support (PP = 0.67). 

For the species tree with species-lineages inferred using sequence data, estimates of divergence times using the four different rate-specific scenarios (fixed rates for the ITS, nrLSU, RPB1, and ITS with nrLSU) are reported in Figure 3A. The substitution rates of the nine sampled loci for each of the four rate-scenarios using the genetic population clusters, estimated under a relaxed clock, are reported in Table 2. In analyses using traditional phenotype-based species, the substitution rates of the nine sampled loci estimated for the two markers resulted in the highest and lowest rates, ITS and RPB1 respectively, are reported in Table 3 In all scenarios, divergence events were estimated to have occurred during the Pleistocene (Figures 3A, B). However, estimates varied widely depending on the specific fixed substitution rate, with the fixed ITS rate resulting in the oldest estimates and the rate for the RPB1 marker yielding the most recent estimates (Figure 3A). Divergence estimates were shown to differ by between 0.6 and 1.6 million years, depending on the specified substitution rate (Figure 3A). For example, the divergence times estimated using the fixed ITS rate support the separation of clades ‘D’ and ‘E’ ca. 2.3 Ma (95% HPD = 1.7–3.0), while this split was estimated to have occurred ca. 0.7 Ma (95% HPD = 0.4–1.0) using the fixed rate for the RPB1 locus (Figure 3A). Divergence estimates indicate that in both clades ‘D’ and ‘E’ the majority of the diversification leading to extant species, including the vagrant species, occurred between 0.3 and 1.9 Ma. 

**Table 3:** Estimated substitution rates for western North American *Xanthoparmelia* clades ‘D’ and ‘E’ from the *BEAST analysis using traditional, phenotype-based species circumscriptions.

	ITS	RPB1
IGS	2.11 (HPD = 1.39 - 2.94)	7.30 (HPD = 4.19 - 10.66)
Intron	2.27 (HPD = 1.48 - 3.17)	7.85 (HPD = 4.53 - 11.62)
nrLSU	0.61 (HPD = 0.36 - 0.88)	2.08 (HPD = 1.10 - 3.16)
ITS	2.39 (fixed 2.43)	9.51 (HPD = 5.60 - 13.75)
BT	1.04 (HPD = 0.66 - 1.44)	3.56 (HPD = 2.03 - 5.25)
GAPDH	1.58 (HPD = 0.97 - 2.27)	5.29 (HPD = 2.96 - 8.03)
MCM7	2.86 (HPD = 1.77 - 4.04)	9.61 (HPD = 5.50 - 14.16)
RPB1	0.60 (HPD = 0.36 - 0.86)	1.55 (fixed = 1.51)
RPB2	1.45 (HPD = 0.93 - 2.02)	4.96 (HPD = 2.89 - 7.37)

Rates were estimated under an uncorrelated lognormal relaxed molecular clock using fixed substitution rates for the two analyses that resulted in the oldest and youngest estimates, the ITS and RPB1 rates, respectively (fixed rates shown in parentheses). Units: substitution/site/10^9^ years.

For the morphology-based species tree, divergence times from the two analyses that resulted in the oldest and youngest estimates, the ITS and RPB1 rates, respectively, are shown in Figure 3B. Similar to divergence times estimated in the genetic cluster-based gene tree, divergence events were also estimated to have occurred during the Pleistocene in the morphology-based species tree. However, age estimates in the morphology-based species tree were more recent than those inferred from the genetic cluster-based gene tree analysis. In the morphology-based tree using the fixed rate for the ITS marker, the initial radiation of species within ‘clade D’ was estimated to have occurred ca. 0.4 Ma (95% HPD = 0.2–0.7) (Figure 3B), but ca. 1.9 Ma (95% HPD = 1.3–2.5) in the cluster-based tree (Figure 3A). Similarly, the initial radiation of ‘clade E’ was estimated at ca. 0.8 Ma (95% HPD = 0.5–1.4) in the morphology-based species tree (Figure 3B), but 1.5 Ma (95% HPD = 1.0–2.0) in the cluster-based tree (Figure 3A).

### Speciation probabilities

Speciation probabilities based on the BP&P analyses are shown in Figures 3A, B. Within ‘clade D’, traditional phenotype based species (based largely on diagnostic extrolites) were not supported in the BP&P analyses (Figure 3B). In contrast, most putative species groups in ‘clade E’ received strong support in both the morphology-based and genetic cluster species delimitation scenarios, with the exception of the vagrant species X. aff. *chlorochroa*, *X. lipochlorochroa*, *X*. aff. *neochlorochroa*, and X. aff. *norchlorochroa* which were collapsed as a single species (Figure 3). Comparing alternative guide trees representing alternative relationships at weakly supported nodes resulted in similar levels of support at those nodes (results not shown).

## Discussion

In this study, we compared two alternative, though somewhat overlapping, species delimitation scenarios in two clades in the lichenized fungal genus *Xanthoparmelia* from western North America. In the first scenario we used Bayesian clustering and phylogenetic inference to identify putative lineages based on molecular sequence data; and the second scenario was based on traditional morphology/chemistry-based species circumscriptions (Table 4). We found that neither genetic clustering nor Bayesian species delimitation supported traditional phenotype-based species in a clade dominated by isidiate morphs (‘clade D’; Figures 1 and 3B). In contrast, the Bayesian species delimitation method implemented in this study generally provided strong support for both the phenotype- and allelic-based species circumscriptions in ‘clade E’. However, divergence estimates differed between the two approaches for delimiting species, demonstrating that differences in phenotype- and molecular-based species circumscriptions may have important implications for estimating divergence times within a coalescent-based framework (Figure 3). Further confounding our ability to assess the timing of species diversification in *Xanthoparmelia*, we found that rates of molecular evolution for different loci, estimated from closely related taxa, resulted in substantial differences in divergence times (Figure 3A,B). In spite of these challenges, our results indicate that the Pleistocene was an important period of diversification for *Xanthoparmelia* in western North America, and this study provides additional insight into species boundaries for this group.

**Table 4:** Provisional taxonomy for montane *Xanthoparmelia* clades ‘D’ and ‘E’ in western North America.

Provisional taxon	Bayesian clustering (see Figure 1)	Phylogeny (see Figure 2)	Traditional species	General comments/notes
‘X. aff. *lineola*’	‘D’	‘Clade D-1’	X. aff. *lineola*	Non-isidiate forms expressing the salazinic acid chemosyndrome, know from three specimens collected in montane habitats in Utah (Duchesne & Washington Counties), USA.
‘*X. isidiomontana*’*	‘D’	‘Clade D-2’	X. aff. *dierythra*, *X*. aff. *lineola*, *X*. aff. *mexicana*, and X. aff. *plitti*)	Lineage dominated by the chemically polymorphic (norstictic, salazinic, and stictic acid chemosyndromes), isidiate forms in continental habitats in the Intermountain Western USA, east to the western Dakotas, USA.
‘*X. subcumberlandia*’	‘E-1a’	‘Custer E-1a’	X. aff. *cumberlandia*	Non-isidiate forms expressing the stictic acid chemosyndrome, from continental habitats in the Intermountain Western USA.
‘*X. utahensis*’*	‘E-1b’	‘Clade E-1b’	X. aff. *coloradoënsis*	Non-isidiate forms expressing the salazinic acid chemosyndrome, from continental habitats in the Intermountain Western USA.
‘*X. wyomingica*’	‘E-2a’, ‘E-2b’, ‘E-2c’	‘Clade E-2’	X. aff. *chlorochroa*, *X*. aff. *coloradoënsis*, *X*. aff. *cumberlandia*, *X*. aff. *lipochlorochroa*, *X*. aff. *neochlorochroa*, *X*. aff. *norchlorochroa*, *X*. aff. *vagans*, and X. aff. *wyomingica*,	Morphologically variable forms, ranging from adnate saxicolous forms to semi-attached and completely vagrant terricolous forms. Saxicolous forms generally express the salazinic acid chemosyndrome, but vagrant forms are chemically polymorphic, producing salazinic, stictic, nortstic, and fatty acid chemosyndromes.

^*^ new putative species

### Equivocal species boundaries and the impact on divergence estimates

 Despite increasing amounts of data and improved perspectives on species boundaries, both species discovery and specimen identification remains challenging [1,105]. In general, lichens display relatively few taxonomically useful characters, and studies have repeatedly shown that our current interpretation of morphological and chemical characters often fails to accurately characterize species diversity [42]. Our study of *Xanthoparmelia* lineages in western North America empirically demonstrated that equivocal taxonomy could potentially have a significant impact on species tree and divergence time estimates where *a priori* species assignments are required. 

We assessed the validity of both phenotype- and genetic-based species circumscriptions in two *Xanthoparmelia* clades by calculating speciation probabilities using the Bayesian species delimitation program BP&P [32]. Although coalescent-based species delimitation methods are likely a step towards improved objectivity and comparability [6], our empirical study of species boundaries in *Xanthoparmelia* indicates that selection between competing hypotheses of species boundaries remains challenging. Below we explore several reasons which may explain the overall support of species in the differing species delimitation scenarios assessed here, particularly in ‘clade E’. 

First, there was some overlap between the phenotype-based species circumscriptions and the genetic clusters (Table 4). For example, the vast majority of specimens identified as X. aff. *cumberlandia* were recovered within genetic cluster ‘E-1a’, although a limited number of specimens identified as X. aff. *cumberlandia* were also assigned membership in cluster ‘E-2a’. Similarly, a large number of vagrant morphs were recovered in two distinct genetic clusters, ‘E-2b’ and ‘E-2c’ (see Figures 1 & S4), supporting the inference of genetic differentiation of vagrants species from attached saxicolous morphs in the morphology-based scenario. Ideally, both genetic and phenotypical data should support similar hypotheses of species boundaries [7]; however, in practice different data sets and operational criteria may give conflicting or ambiguous results [106-109]. Our empirical study of *Xanthoparmelia* species supports the potential for strong speciation probabilities in differing species delimitation scenarios in cases where species hypotheses based on phenotypical characters and genetic data overlap to some degree. 

Second, in the phenotype-based species delimitation scenario X. aff. *norchlorochroa* and *X. wyomingica* were represented by only two individuals. Previous studies suggest that this sampling density (both in number of individuals and loci) may be adequate to accurately infer speciation probabilities, even in cases of recent divergence [33,100]. However, *X. wyomingica* sensu lato (s.l.) often occurs in intermediate morphs, integrating between nearly completely vagrant forms – similar to *X. chlorochroa* s.l. – to loosely attached forms – similar to *X. coloradoënsis* s.l. [74,110]. Our study included only two characteristic specimens collected from a single population near the type locality in the Bighorn Mountains, Wyoming, USA. Including additional *X. wyomingica* s.l. specimens from other populations, including those with deviating morphologies, could potentially have a substantial impact on our inference of species boundaries. 

A third reason that may have contributed to the general support for competing species delimitation hypotheses is the exclusion of individuals with evidence of admixed ancestry. Although some degree of gene flow between incipient species may be a common phenomenon [13,111], coalescent-based approaches do not account for this source of gene-tree discordance [21,96,112]. In order to mitigate potentially confounding effects of gene flow on species tree inference, sampling schemes tend to avoid including individuals from phylogeographic lineage boundaries or individuals with evidence of admixed ancestry [48,96]. While eliminating individuals affected by introgression may improve accuracy of species tree reconstructions, the removal of individuals showing evidence of an admixed genome likely has a significant impact on speciation probabilities by inflating the perceived amount of genetic divergence among populations.

The exclusion of specimens with admixed ancestries in BP&P is largely a practical issue when individual population assignments are equivocal or if there is evidence of hybridization between two distinct populations (ex. hybrid boundary, intermediate morphologies, etc.), thereby violating the assumption of complete cessation of gene flow following species divergence [32,100]. As expected, the accuracy of BP&P has been shown to decrease with increasing levels of interspecific introgression, although the program still performs relatively well using a modest number of loci (4-10) and individuals per species, despite low levels of gene flow [21,100]. BP&P has been shown to be indecisive at intermediate levels of migration and tends to lump populations into a single species at higher migration rates (> 10 migrants per generation) [100]. 

The exclusion of individuals with ambiguous population membership in groups where population boundaries are not clear likely has a substantial impact on estimating speciation probabilities. In our study of montane *Xanthoparmelia* populations in western North America, ca. 20% of the individuals within ‘cluster E-2’ were inferred with admixed ancestry in the STRUCTURE analyses (see Figure 1) and were excluded from the BP&P analysis. The admixed individuals included characteristic forms of X. aff. *chlorochroa*, *X*. aff. *coloradoënsis*, and *X. wyomingica*, and none of these specimens displayed any phenotypical evidence of hybridization. The specimens with inferred admixed ancestry may simply be an artifact of our limited molecular sampling or other methodological limitations, rather than actually representing truly admixed individuals. Ultimately, the treatment of individuals with inferred admixed ancestry must be carefully considered on a case-by-case basis. 

Potential violations of assumptions may also limit the practical application of speciation probabilities estimated in BP&P [5,32,100]. BP&P requires the input of a user-specified guide tree and misspecification of the guide tree can result in strong support for models containing more species by artificially increasing divergence among populations [5]. In our study of *Xanthoparmelia*, we compared different guide trees representing alternative topologies in cases where relationships were not strongly supported in the coalescent-based species tree and found similar speciation probabilities in all cases (data not shown). Therefore, we assert that the high speciation probabilities observed in this study are likely not the result of a mis-specified guide tree. Prior distributions for ancestral population sizes (θ) and the root age (τ_0_) may also have a significant impact on species delimitation results [5,32]. In many cases, including *Xanthoparmelia*, little is known of ancestral population sizes and root age, requires a largely subjective specification for θ and τ_0._ In general, a combination of large θ and small τ_0_ provide a more conservative estimate supporting fewer species, although species delimitation results appear to be robust with changes to τ_0_ [5,32]. For species delimitation in *Xanthoparmelia* we assumed intermediate ancestral population sizes and relatively shallow divergences among species (see Methods), but speciation probabilities were similar under a more conservative combination of priors, 0.1 and 0.001 for θ and τ_0_, respectively (data not shown).

Given general support for both species delimitation scenarios, we assessed divergence estimates for both hypotheses within a coalescent-based multispecies species tree approach. Our results revealed some differences in estimates of divergence times. Divergence time estimates of the initial split between clades ‘E’ and ‘D’ were consistent for both morphology- and genetic-based species trees, depending on the specified rate calibration (Figures 3A, B). However, differences in divergence time estimates between morphology- and molecular-based species trees become apparent in clades ‘D’ and ‘E’, with non-overlapping HPD intervals for the initial radiation of ‘clade D’(Figures 3A, B). Estimates for the initial radiation of ‘clade E’ differed by up to 0.7 million years, 1.5 Ma in the population cluster-based species tree and 0.8 Ma in the phenotype-based species tree, although HPD intervals overlapped between 1.0 - 1.3 Ma (Figures 3A, B). The potential for discrepancy among divergence estimates from ambiguous species delimitation hypotheses has important implications for inferring specific factors driving diversification in western North American lichens, particularly in more recent diversification scenarios such as we see in western North American *Xanthoparmelia* species. 

 In this study, we used a number of genetic markers that have commonly been implemented to assess relationships among closely related species, and intraspecific evolutionary processes [67,80,113-115]. However, morphology and genetic-based *Xanthoparmelia* lineages showed strong evidence of incomplete lineage sorting across all sampled loci. Qualitatively, the RPB1 gene tree (Figure S3) was most similar to the 9-gene total evidence phylogeny and the 5-gene protein-coding phylogeny (Figures S4, S6). This finding is consistent with other studies highlighting the utility of the RPB1 for accurately inferring relationships at multiple scales [78,116,117]. In contrast, relationships in the four-gene ribosomal phylogeny generally lacked support and did not correspond to either morphology- or genetic-based lineages (Figure S5). Overall, our results highlight the pressing need to develop additional markers in order to resolve species relationships in montane *Xanthoparmelia* in western North America with greater confidence.

### The impact of substitution rates on divergence estimates

 Our results show that using mutation rates estimated for different loci may result in more substantial differences in divergence estimates than those inferred using alternative species delimitation scenarios in the sampled *Xanthoparmelia* lineages (Figure 3). In the absence of relevant fossil evidence for *Xanthoparmelia*, we assessed divergence times using Parmeliaceae-specific mutation rates from three loci, ITS, nrLSU, and RPB1 [45,58]. Divergence estimates using substitution rates for the ITS, nrLSU, and the combination of ITS/nrLSU loci resulted in at least partially overlapping highest posterior density (HPD) regions, but differed significantly from estimates using the substitution rate for the RPB1 marker (Figure 3). Previous studies have shown relatively consistent substitution rates (ca. 1.5 substitution/site/year × 10^-9^) for a number of protein coding loci, including MCM7, RPB1, RPB2, across multiple genera in Parmeliaceae [25,45]. However, estimated substitution rates for protein-coding loci used in this study varied widely, with the RPB1 marker showing markedly slower mutation rates, when compared to the MCM7 and RPB2 markers (Tables 2 & 3). Amo de Paz et al. [58] also showed that substitution rates for the RPB1 marker varied widely among major lineages in Parmeliaceae. In contrast, estimates of ITS substitution rates for a range of lichen-forming mycobionts and a non-lichenized fungus were relatively similar [25,45,68,101]. Additional research will be required to better understand differences in evolutionary rates across loci, lineages, and different temporal scales in order to identify loci that may provide more reliable rate-calibrated divergence estimates [118-120]. However, in the absence of fossil calibration points, an exploration of all reasonable substitution rates from different loci will be important in showing a more complete range of potential divergence times. 

### Pleistocene-driven speciation in Xanthoparmelia in western North America

Our study indicates that Pleistocene glacial cycles played a major role in *Xanthoparmelia* diversification in montane habitats in western North America. Although alternative species hypotheses and rates of molecular evolution for different loci resulted in varying and occasionally non-overlapping divergence times (Figure 3), diversification events are consistently estimated to have occurred during the Pleistocene in the two *Xanthoparmelia* clades investigated here (clades ‘D’ and ‘E’; see 48. Recent studies have also revealed previously unrecognized species-level divergence coinciding with Pleistocene climatic fluctuations in the *Rhizoplaca melanophthalma* species-complex (Lecanoraceae) [64] and two species complexes in the genus *Melanohalea* (Parmeliaceae), *M. elegantula* s.l. and *M. subolivacea* s.l. [25]. Interestingly, *Rhizoplaca melanophthalma* s.l. and *Melanohalea subolivacea* s.l. often co-occur to varying degrees with the sampled *Xanthoparmelia* lineages across a broad range of habitats in western North America, ranging from pinyon-juniper woodlands into subalpine and even alpine habitats. The shared temporal diversification pattern in these lichen-forming fungal groups suggests that speciation may have been driven by similar factors during the Pleistocene glaciations in western North America. 

Subtle differences in microhabitats have been shown to have a strong impact on the occurrence and distribution of some lichens [121-124], and ecological processes may be central to the formation of new species through ecology-based divergent selection [125,126]. However, there is little evidence of ecological divergence among the closely related *Xanthoparmelia* lineages found in both clades ‘D’ and ‘E’. Most species-level lineages were found across a broad range of habitats and were commonly found occurring in sympatry at local scales [127]. Similarly, distinct lineages within the *Rhizoplaca melanophthalma* and *Melanohalea subolivacea* species complexes showed no apparent preference for distinct environmental conditions [25,64,88]. Ultimately, empirically assessing environmental parameters and modeling ecological niches may provide important insights into the specific processes driving speciation in these groups. 

The biological roles of the different medullary compounds remain uncertain [128-130]. Medullary extrolites have traditionally played a major role in *Xanthoparmelia* taxonomy [74]. However, our results indicate that differences in secondary chemistry in isidiate morphs in ‘clade D’ and vagrant morphs in ‘clade E’ do not correspond with distinct evolutionary lineages (Table 4). In contrast, saxicolous forms in ‘clade E’ producing stictic acid are supported by molecular data as a lineage distinct from morphologically similar forms producing salazinic acid (Table 4). Differences in morphology and chemistry across substrata and habitats have been documented for *Xanthoparmelia* populations in the Sonoran Desert [131], and additional research may provide novel insights into the role of distinct extrolites on the fitness of *Xanthoparmelia* species. 

 Although species boundaries in the sampled *Xanthoparmelia* clades remain somewhat equivocal, these data provide valuable insights for improving our perspective concerning the species boundaries of montane *Xanthoparmelia* in western North America. A number of recent studies have demonstrated that some morphological and chemical characters traditionally used to separate species likely represent intraspecific polymorphisms rather than taxonomically diagnostic traits [47,49,132,133]. Our population clustering analysis supports a broader phenotypic circumscription for the *Xanthoparmelia* lineages sampled here. Based on the results from the Bayesian clustering and phylogenetic analyses and general patterns in morphological and/or chemical characters, we propose an alternative hypothesis of species boundaries within the sample clades and recommend a number of provisional names (Table 4). A complete taxonomic revision of species within clades ‘D’ and ‘E’ will be treated in detail in a companion paper (Leavitt et al. *in prep*). 

## Supporting Information

Figure S1 **Population assignments to genetic clusters in *Xanthoparmelia* ‘clade D’.** Population membership was inferred using the Program BAPS and STRUCTURE using SNP data from nine sampled loci (nrLSU, IGS, ITS, group I intron, β-tubulin, GAPDH, MCM7, RPB1, and RPB2). STRUCUTRE analyses include estimates for *K* = 2 and *K* = 4 models; and the BAPS analyses included the estimated number of clusters (*K* = 1). Each accession is shown by a thin vertical line that is partitioned into colored segments representing the proportion of each individual’s genome assigned to a genetic cluster.
(PDF)

Figure S2 **Evaluation of the STRUCTURE results from the *Xanthoparmelia* ‘clade E’.** SNP data from nine sampled loci (nrLSU, IGS, ITS, group I intron, β-tubulin, GAPDH, MCM7, RPB1, and RPB2), ribosomal loci only (nrLSU, IGS, ITS, group I intron) and protein-coding loci only (β-tubulin, GAPDH, MCM7, RPB1, and RPB2) were included. (A) Likelihood values estimated from ten independent runs from each *K* from 1 - 6. (B) Results of the Δ*K* method for inferring the number of distinct population clusters.
(PDF)

Figure S3 **Maximum likelihood gene trees for *Xanthoparmelia* clades ‘D’ and ‘E’.** Topologies were estimated from each of the nine sampled loci (nrLSU, IGS, ITS, group I intron, β-tubulin, GAPDH, MCM7, RPB1, and RPB2), with bootstrap support indicated at nodes. Colors in ‘clade E’ correspond to genetic clusters inferred from the *K* = 5 model; colors in ‘clade D’ correspond to two well-supported clades recovered in the concatenated multilocus phylogeny (see Figure S4). Tip labels include the individual STRUCTURE assignment for the *K* = 2 (*K* = 1 for ‘clade D’), *K* = 5 (*K* = 4 for ‘clade D’), morphological identification, and specimen number, in that order.
(PDF)

Figure S4 **Total evidence ML tree estimated from the concatenated nine loci data matrix.** Loci included: nrLSU, IGS, ITS, group I intron, β-tubulin, GAPDH, MCM7, RPB1, and RPB2; and bootstrap support indicated at nodes. Colors in ‘clade E’ correspond to genetic clusters inferred from the *K* = 5 model; colors in ‘clade D’ correspond to two well-supported clades recovered in this total evidence phylogeny.
(PDF)

Figure S5 **ML tree estimated from the concatenated ribosomal loci.** Loci include: nrLSU, IGS, ITS, and group I intron; and bootstrap support indicated at nodes. Colors in ‘clade E’ correspond to genetic clusters inferred from the *K* = 5 model; colors in ‘clade D’ correspond to two well-supported clades recovered in the concatenated multilocus phylogeny (see Figure S4).
(PDF)

Figure S6 **ML tree estimated from the concatenated protein-coding loci.** Loci include: β-tubulin, GAPDH, MCM7, RPB1, and RPB2; and bootstrap support indicated at nodes. Colors in ‘clade E’ correspond to genetic clusters inferred from the *K* = 5 model; colors in ‘clade D’ correspond to two well-supported clades recovered in the concatenated multilocus phylogeny (see Figure S4).
(PDF)

Table S1 **Collection information for all *Xanthoparmelia* specimens included in the present study** : ID, individual code; morphological/chemical species identification; inferred population cluster; Brigham Young University Herbarium of Non-vascular Cryptogams (BRY) voucher accession number; major acid, diagnostic secondary chemistry; Location; Lat., latitude; Lon., longitude; Ele., altitude in meters a.s.l.; collector(s), and GenBank accession numbers.
(XLSX)
