# Supplementary figures and images for: Pleistocene Speciation in North American Lichenized Fungi and the Impact of Alternative Species Circumscriptions and Rates of Molecular Evolution on Divergence Estimates
Source: PLoS One. 2013 Dec 26;8(12):e85240. doi: 10.1371/journal.pone.0085240 (PMC3873437; doi:10.1371/journal.pone.0085240)

**BAPS  $K = \text{estimated}$**

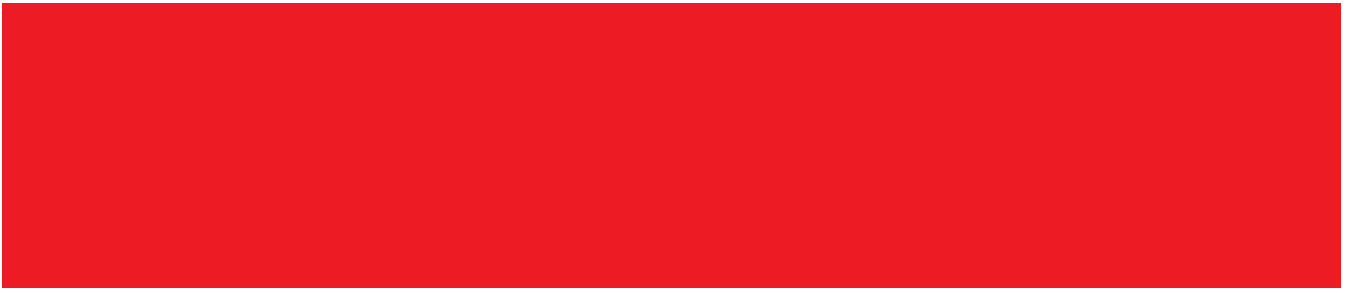

**STRUCTURE:  $K = 2$**

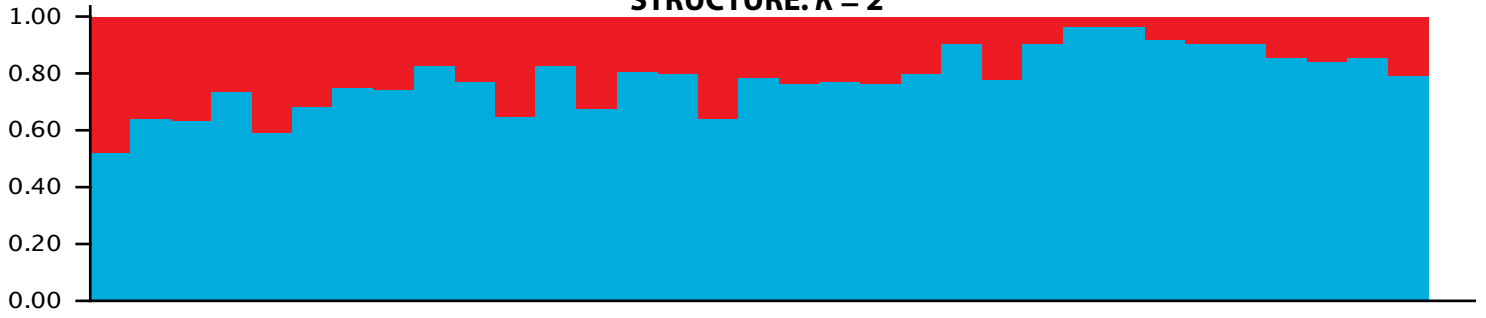

**STRUCTURE:  $K = 4$**

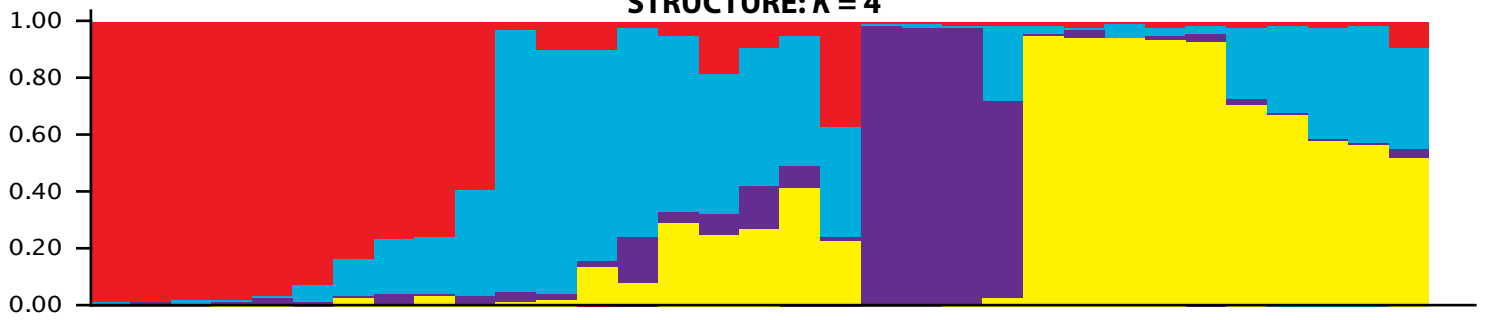

Supplement: Figure S1 — Population assignments to genetic clusters in Xanthoparmelia ‘clade D’. Population membership was inferred using the Program BAPS and STRUCTURE using SNP data from nine sampled loci (nrLSU, IGS, ITS, group I intron, β-tubulin, GAPDH, MCM7, RPB1, and RPB2). STRUCUTRE analyses include estimates for K = 2 and K = 4 models; and the BAPS analyses included the estimated number of clusters (K = 1). Each accession is shown by a thin vertical line that is partitioned into colored segments representing the proportion of each individual’s genome assigned to a genetic cluster. (PDF) [file pone.0085240.s001.pdf]

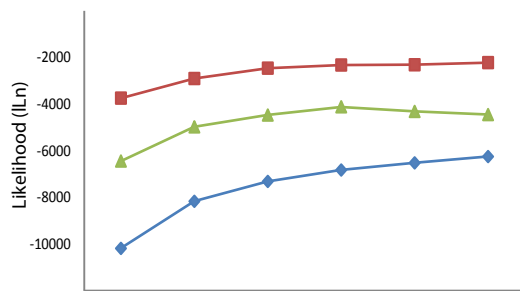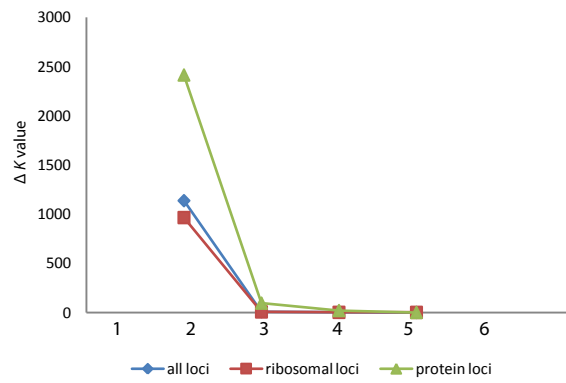

Supplement: Figure S2 — Evaluation of the STRUCTURE results from the Xanthoparmelia ‘clade E’. SNP data from nine sampled loci (nrLSU, IGS, ITS, group I intron, β-tubulin, GAPDH, MCM7, RPB1, and RPB2), ribosomal loci only (nrLSU, IGS, ITS, group I intron) and protein-coding loci only (β-tubulin, GAPDH, MCM7, RPB1, and RPB2) were included. (A) Likelihood values estimated from ten independent runs from each K from 1 - 6. (B) Results of the ΔK method for inferring the number of distinct population clusters. (PDF) [file pone.0085240.s002.pdf]

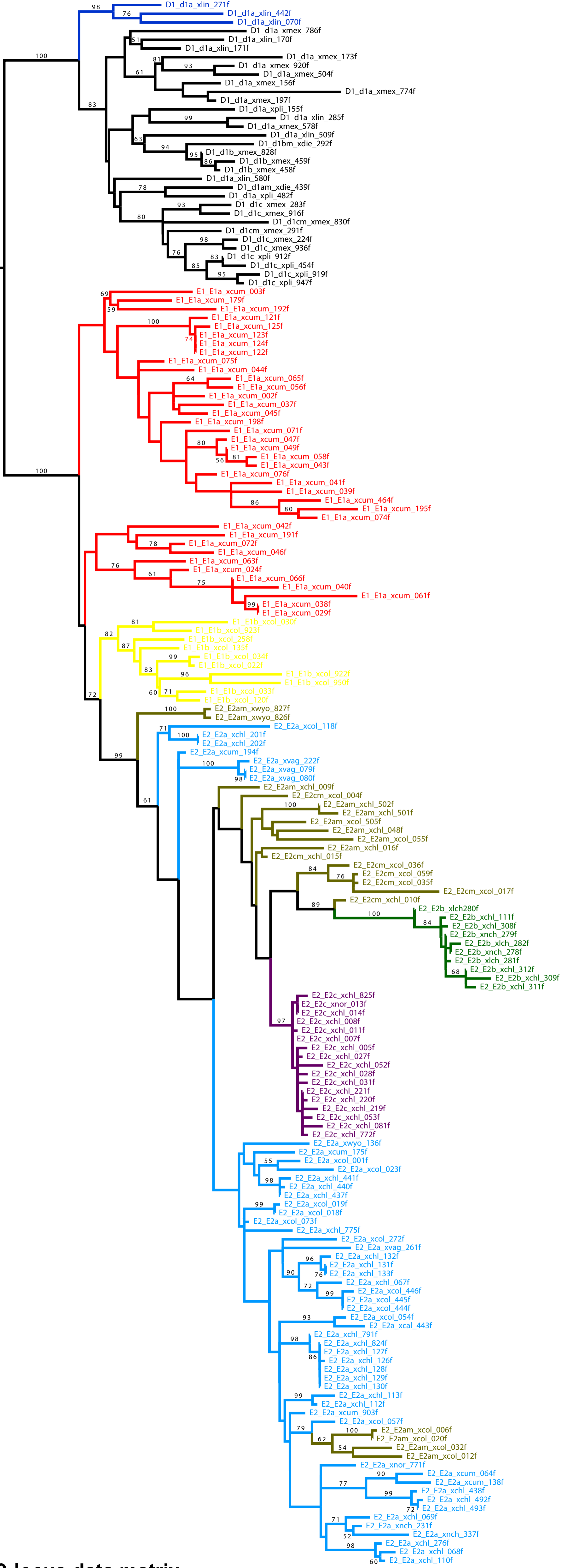

# 9-locus data matrix

ITS, nrLSU, IGS, intron,  $\beta$ -tubulin, GAPDH, MCM7, RPB1, RPB2

0.0070 substitutions/site

Supplement: Figure S4 — Total evidence ML tree estimated from the concatenated nine loci data matrix. Loci included: nrLSU, IGS, ITS, group I intron, β-tubulin, GAPDH, MCM7, RPB1, and RPB2; and bootstrap support indicated at nodes. Colors in ‘clade E’ correspond to genetic clusters inferred from the K = 5 model; colors in ‘clade D’ correspond to two well-supported clades recovered in this total evidence phylogeny. (PDF) [file pone.0085240.s004.pdf]

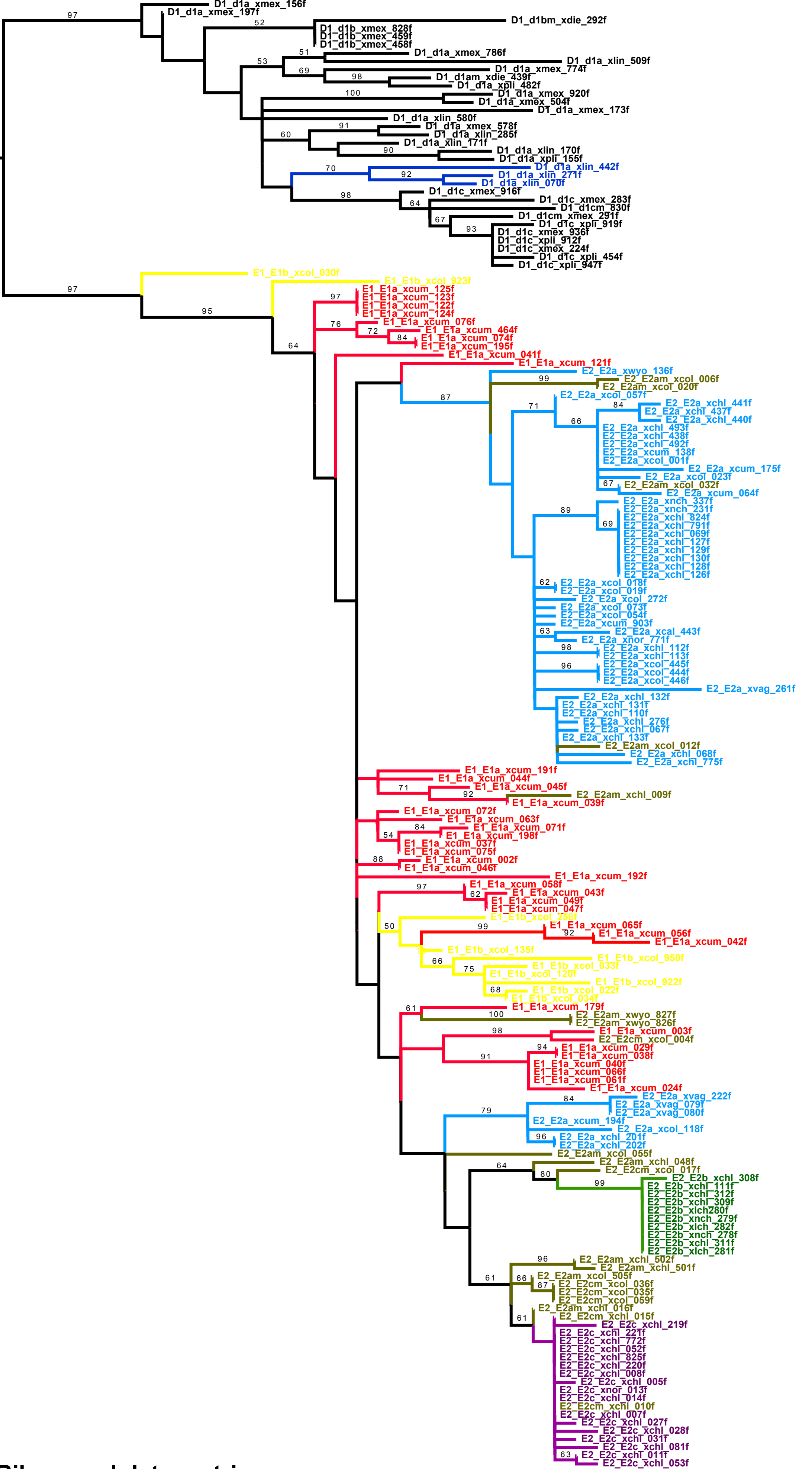

Ribosomal data matrix

ITS, nrLSU, IGS, intron

0.0030 substitutions/site

Supplement: Figure S5 — ML tree estimated from the concatenated ribosomal loci. Loci include: nrLSU, IGS, ITS, and group I intron; and bootstrap support indicated at nodes. Colors in ‘clade E’ correspond to genetic clusters inferred from the K = 5 model; colors in ‘clade D’ correspond to two well-supported clades recovered in the concatenated multilocus phylogeny (see Figure S4). (PDF) [file pone.0085240.s005.pdf]

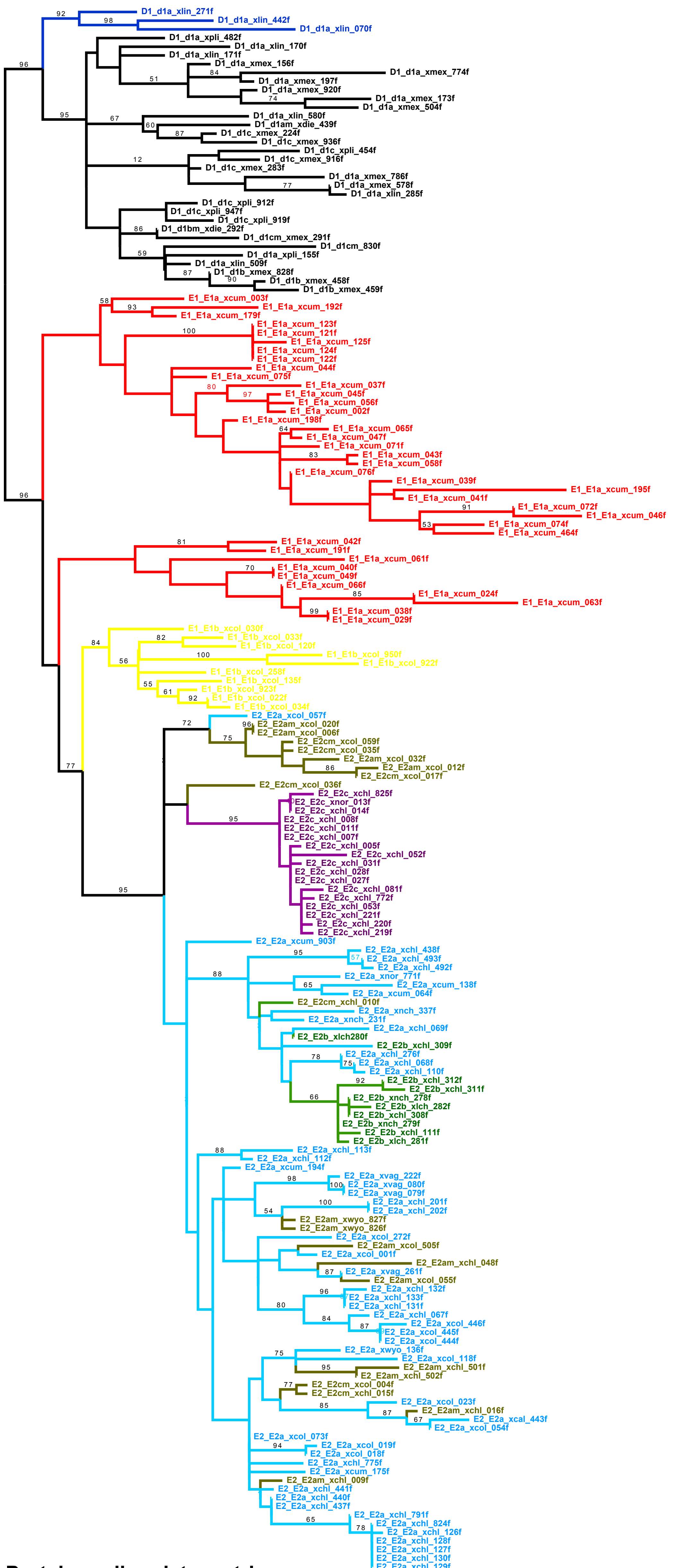

Supplement: Figure S6 — ML tree estimated from the concatenated protein-coding loci. Loci include: β-tubulin, GAPDH, MCM7, RPB1, and RPB2; and bootstrap support indicated at nodes. Colors in ‘clade E’ correspond to genetic clusters inferred from the K = 5 model; colors in ‘clade D’ correspond to two well-supported clades recovered in the concatenated multilocus phylogeny (see Figure S4). (PDF) [file pone.0085240.s006.pdf]
