# Supplementary material for: Pleistocene Speciation in North American Lichenized Fungi and the Impact of Alternative Species Circumscriptions and Rates of Molecular Evolution on Divergence Estimates
Source: PLoS One. 2013 Dec 26;8(12):e85240. doi: 10.1371/journal.pone.0085240 (PMC3873437; doi:10.1371/journal.pone.0085240)
Supplement: Figure S3 — Maximum likelihood gene trees for Xanthoparmelia clades ‘D’ and ‘E’. Topologies were estimated from each of the nine sampled loci (nrLSU, IGS, ITS, group I intron, β-tubulin, GAPDH, MCM7, RPB1, and RPB2), with bootstrap support indicated at nodes. Colors in ‘clade E’ correspond to genetic clusters inferred from the K = 5 model; colors in ‘clade D’ correspond to two well-supported clades recovered in the concatenated multilocus phylogeny (see Figure S4). Tip labels include the individual STRUCTURE assignment for the K = 2 (K = 1 for ‘clade D’), K = 5 (K = 4 for ‘clade D’), morphological identification, and specimen number, in that order. (PDF) [file pone.0085240.s003.pdf]

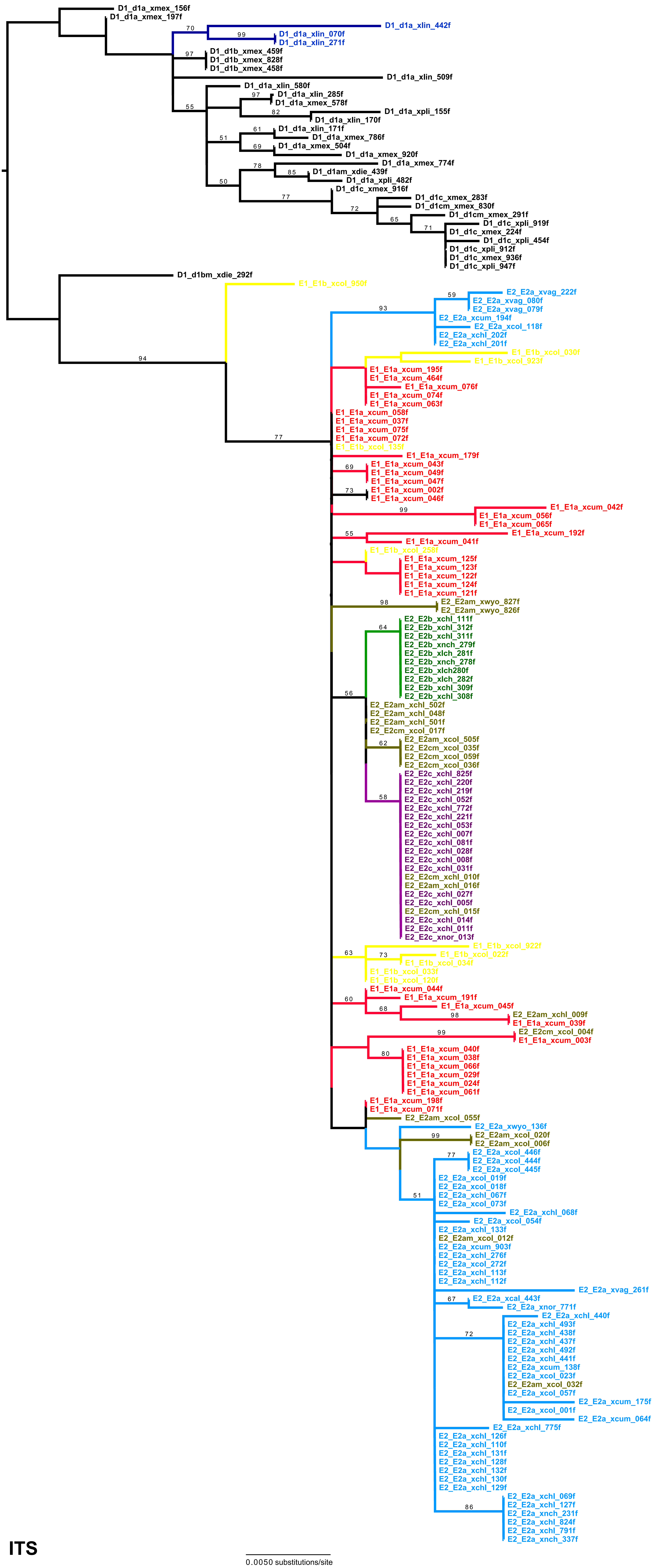

D1_d1a_xmex_156f
D1_d1a_xmex_197f
70
D1_d1a_xlin_442f
99
D1_d1a_xlin_070f
D1_d1a_xlin_271f
97
D1_d1b_xmex_459f
D1_d1b_xmex_828f
D1_d1b_xmex_458f
D1_d1a_xlin_509f
D1_d1a_xlin_580f
97
D1_d1a_xlin_285f
55
D1_d1a_xmex_578f
82
D1_d1a_xpli_155f
D1_d1a_xlin_170f
61
D1_d1a_xlin_171f
51
D1_d1a_xmex_786f
69
D1_d1a_xmex_504f
D1_d1a_xmex_920f
78
D1_d1a_xmex_774f
85
D1_d1am_xdie_439f
50
D1_d1a_xpli_482f
D1_d1c_xmex_916f
77
D1_d1c_xmex_283f
72
D1_d1cm_xmex_830f
65
D1_d1cm_xmex_291f
71
D1_d1c_xpli_919f
D1_d1c_xmex_224f
D1_d1c_xpli_454f
D1_d1c_xpli_912f
D1_d1c_xmex_936f
D1_d1c_xpli_947f
D1_d1bm_xdie_292f
E1_E1b_xcol_950f
E2_E2a_xvag_222f
59
E2_E2a_xvag_080f
93
E2_E2a_xvag_079f
E2_E2a_xcum_194f
E2_E2a_xcol_118f
E2_E2a_xchl_202f
E2_E2a_xchl_201f
E1_E1b_xcol_030f
94
E1_E1b_xcol_923f
E1_E1a_xcum_195f
E1_E1a_xcum_464f
E1_E1a_xcum_076f
E1_E1a_xcum_074f
E1_E1a_xcum_063f
E1_E1a_xcum_058f
E1_E1a_xcum_037f
E1_E1a_xcum_075f
77
E1_E1a_xcum_072f
E1_E1b_xcol_135f
E1_E1a_xcum_179f
69
E1_E1a_xcum_043f
E1_E1a_xcum_049f
E1_E1a_xcum_047f
73
E1_E1a_xcum_002f
E1_E1a_xcum_046f
E1_E1a_xcum_042f
99
E1_E1a_xcum_056f
E1_E1a_xcum_065f
55
E1_E1a_xcum_192f
E1_E1a_xcum_041f
E1_E1b_xcol_250f
E1_E1a_xcum_125f
E1_E1a_xcum_123f
E1_E1a_xcum_122f
E1_E1a_xcum_124f
E1_E1a_xcum_121f
98
E2_E2am_xwyo_827f
E2_E2am_xwyo_826f
E2_E2b_xchl_111f
64
E2_E2b_xchl_312f
E2_E2b_xchl_311f
E2_E2b_xnch_279f
E2_E2b_xlch_281f
E2_E2b_xnch_278f
E2_E2b_xlch280f
E2_E2b_xlch_282f
56
E2_E2b_xchl_309f
E2_E2b_xchl_308f
E2_E2am_xchl_502f
E2_E2am_xchl_048f
E2_E2am_xchl_501f
E2_E2cm_xcol_017f
E2_E2am_xcol_505f
62
E2_E2cm_xcol_035f
E2_E2cm_xcol_059f
E2_E2cm_xcol_036f
E2_E2c_xchl_825f
E2_E2c_xchl_220f
E2_E2c_xchl_219f
58
E2_E2c_xchl_052f
E2_E2c_xchl_772f
E2_E2c_xchl_221f
E2_E2c_xchl_053f
E2_E2c_xchl_007f
E2_E2c_xchl_081f
E2_E2c_xchl_028f
E2_E2c_xchl_008f
E2_E2c_xchl_031f
E2_E2cm_xchl_010f
E2_E2am_xchl_016f
E2_E2c_xchl_027f
E2_E2c_xchl_005f
E2_E2cm_xchl_015f
E2_E2c_xchl_014f
E2_E2c_xchl_011f
E2_E2c_xnor_013f
E1_E1b_xcol_922f
63
73
E1_E1b_xcol_022f
E1_E1b_xcol_034f
E1_E1b_xcol_033f
E1_E1b_xcol_120f
E1_E1a_xcum_044f
60
E1_E1a_xcum_191f
68
E1_E1a_xcum_045f
98
E2_E2am_xchl_009f
E1_E1a_xcum_039f
99
E2_E2cm_xcol_004f
E1_E1a_xcum_003f
80
E1_E1a_xcum_040f
E1_E1a_xcum_038f
E1_E1a_xcum_066f
E1_E1a_xcum_029f
E1_E1a_xcum_024f
E1_E1a_xcum_061f
E1_E1a_xcum_198f
E1_E1a_xcum_071f
E2_E2am_xcol_055f
E2_E2a_xwyo_136f
99
E2_E2am_xcol_020f
E2_E2am_xcol_006f
77
E2_E2a_xcol_446f
E2_E2a_xcol_444f
E2_E2a_xcol_445f
E2_E2a_xcol_019f
E2_E2a_xcol_018f
51
E2_E2a_xchl_067f
E2_E2a_xcol_073f
E2_E2a_xchl_068f
E2_E2a_xcol_054f
E2_E2a_xchl_133f
E2_E2am_xcol_012f
E2_E2a_xcum_903f
E2_E2a_xchl_276f
E2_E2a_xcol_272f
E2_E2a_xchl_113f
E2_E2a_xchl_112f
E2_E2a_xvag_261f
67
E2_E2a_xcal_443f
E2_E2a_xnor_771f
E2_E2a_xchl_440f
72
E2_E2a_xchl_493f
E2_E2a_xchl_438f
E2_E2a_xchl_437f
E2_E2a_xchl_492f
E2_E2a_xchl_441f
E2_E2a_xcum_138f
E2_E2a_xcol_023f
E2_E2am_xcol_032f
E2_E2a_xcol_057f
E2_E2a_xcum_175f
E2_E2a_xcol_001f
E2_E2a_xcum_064f
E2_E2a_xchl_775f
E2_E2a_xchl_126f
E2_E2a_xchl_110f
E2_E2a_xchl_131f
E2_E2a_xchl_128f
E2_E2a_xchl_132f
E2_E2a_xchl_130f
E2_E2a_xchl_129f
E2_E2a_xchl_069f
86
E2_E2a_xchl_127f
E2_E2a_xnch_231f
E2_E2a_xchl_824f
E2_E2a_xchl_791f
E2_E2a_xnch_337f
ITS
0.0050 substitutions/site

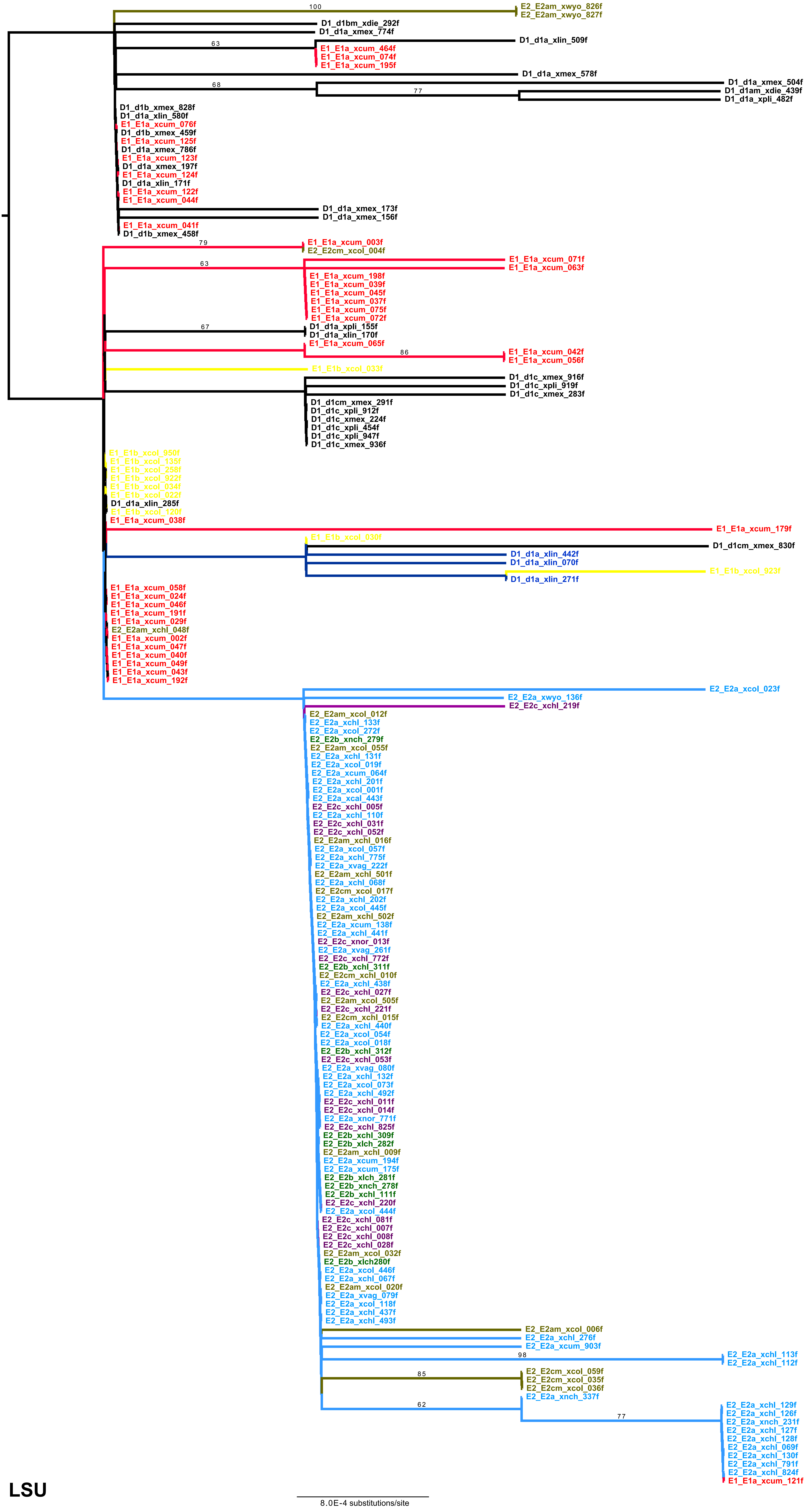

100
E2_E2am_xwyo_826f
E2_E2am_xwyo_827f
D1_d1bm_xdie_292f
D1_d1a_xmex_774f
63
D1_d1a_xlin_509f
E1_E1a_xcum_464f
E1_E1a_xcum_074f
E1_E1a_xcum_195f
D1_d1a_xmex_578f
68
D1_d1a_xmex_504f
77
D1_d1am_xdie_439f
D1_d1a_xpli_482f
D1_d1b_xmex_828f
D1_d1a_xlin_580f
E1_E1a_xcum_076f
D1_d1b_xmex_459f
E1_E1a_xcum_125f
D1_d1a_xmex_786f
E1_E1a_xcum_123f
D1_d1a_xmex_197f
E1_E1a_xcum_124f
D1_d1a_xlin_171f
E1_E1a_xcum_122f
E1_E1a_xcum_044f
D1_d1a_xmex_173f
D1_d1a_xmex_156f
E1_E1a_xcum_041f
D1_d1b_xmex_458f
79
E1_E1a_xcum_003f
E2_E2cm_xcol_004f
63
E1_E1a_xcum_071f
E1_E1a_xcum_063f
E1_E1a_xcum_198f
E1_E1a_xcum_039f
E1_E1a_xcum_045f
E1_E1a_xcum_037f
E1_E1a_xcum_075f
E1_E1a_xcum_072f
67
D1_d1a_xpli_155f
D1_d1a_xlin_170f
E1_E1a_xcum_065f
86
E1_E1a_xcum_042f
E1_E1a_xcum_056f
E1_E1b_xcol_033f
D1_d1c_xmex_916f
D1_d1c_xpli_919f
D1_d1c_xmex_283f
D1_d1cm_xmex_291f
D1_d1c_xpli_912f
D1_d1c_xmex_224f
D1_d1c_xpli_454f
D1_d1c_xpli_947f
D1_d1c_xmex_936f
E1_E1b_xcol_950f
E1_E1b_xcol_135f
E1_E1b_xcol_258f
E1_E1b_xcol_922f
E1_E1b_xcol_034f
E1_E1b_xcol_022f
D1_d1a_xlin_285f
E1_E1b_xcol_120f
E1_E1a_xcum_038f
E1_E1a_xcum_179f
E1_E1b_xcol_030f
D1_d1cm_xmex_830f
D1_d1a_xlin_442f
D1_d1a_xlin_070f
E1_E1b_xcol_923f
D1_d1a_xlin_271f
E1_E1a_xcum_058f
E1_E1a_xcum_024f
E1_E1a_xcum_046f
E1_E1a_xcum_191f
E1_E1a_xcum_029f
E2_E2am_xchl_048f
E1_E1a_xcum_002f
E1_E1a_xcum_047f
E1_E1a_xcum_040f
E1_E1a_xcum_049f
E1_E1a_xcum_043f
E1_E1a_xcum_192f
E2_E2a_xcol_023f
E2_E2a_xwyo_136f
E2_E2c_xchl_219f
E2_E2am_xcol_012f
E2_E2a_xchl_133f
E2_E2a_xcol_272f
E2_E2b_xnch_279f
E2_E2am_xcol_055f
E2_E2a_xchl_131f
E2_E2a_xcol_019f
E2_E2a_xcum_064f
E2_E2a_xchl_201f
E2_E2a_xcol_001f
E2_E2a_xcal_443f
E2_E2c_xchl_005f
E2_E2a_xchl_110f
E2_E2c_xchl_031f
E2_E2c_xchl_052f
E2_E2am_xchl_016f
E2_E2a_xcol_057f
E2_E2a_xchl_775f
E2_E2a_xvag_222f
E2_E2am_xchl_501f
E2_E2a_xchl_068f
E2_E2cm_xcol_017f
E2_E2a_xchl_202f
E2_E2a_xcol_445f
E2_E2am_xchl_502f
E2_E2a_xcum_138f
E2_E2a_xchl_441f
E2_E2c_xnor_013f
E2_E2a_xvag_261f
E2_E2c_xchl_772f
E2_E2b_xchl_311f
E2_E2cm_xchl_010f
E2_E2a_xchl_438f
E2_E2c_xchl_027f
E2_E2am_xcol_505f
E2_E2c_xchl_221f
E2_E2cm_xchl_015f
E2_E2a_xchl_440f
E2_E2a_xcol_054f
E2_E2a_xcol_018f
E2_E2b_xchl_312f
E2_E2c_xchl_053f
E2_E2a_xvag_080f
E2_E2a_xchl_132f
E2_E2a_xcol_073f
E2_E2a_xchl_492f
E2_E2c_xchl_011f
E2_E2c_xchl_014f
E2_E2a_xnor_771f
E2_E2c_xchl_825f
E2_E2b_xchl_309f
E2_E2b_xlch_282f
E2_E2am_xchl_009f
E2_E2a_xcum_194f
E2_E2a_xcum_175f
E2_E2b_xlch_281f
E2_E2b_xnch_278f
E2_E2b_xchl_111f
E2_E2c_xchl_220f
E2_E2a_xcol_444f
E2_E2c_xchl_081f
E2_E2c_xchl_007f
E2_E2c_xchl_008f
E2_E2c_xchl_028f
E2_E2am_xcol_032f
E2_E2b_xlch280f
E2_E2a_xcol_446f
E2_E2a_xchl_067f
E2_E2am_xcol_020f
E2_E2a_xvag_079f
E2_E2a_xcol_118f
E2_E2a_xchl_437f
E2_E2a_xchl_493f
E2_E2am_xcol_006f
E2_E2a_xchl_276f
E2_E2a_xcum_903f
98
E2_E2a_xchl_113f
E2_E2a_xchl_112f
85
E2_E2cm_xcol_059f
E2_E2cm_xcol_035f
E2_E2cm_xcol_036f
E2_E2a_xnch_337f
62
77
E2_E2a_xchl_129f
E2_E2a_xchl_126f
E2_E2a_xnch_231f
E2_E2a_xchl_127f
E2_E2a_xchl_128f
E2_E2a_xchl_069f
E2_E2a_xchl_130f
E2_E2a_xchl_791f
E2_E2a_xchl_824f
E1_E1a_xcum_121f
LSU
8.0E-4 substitutions/site

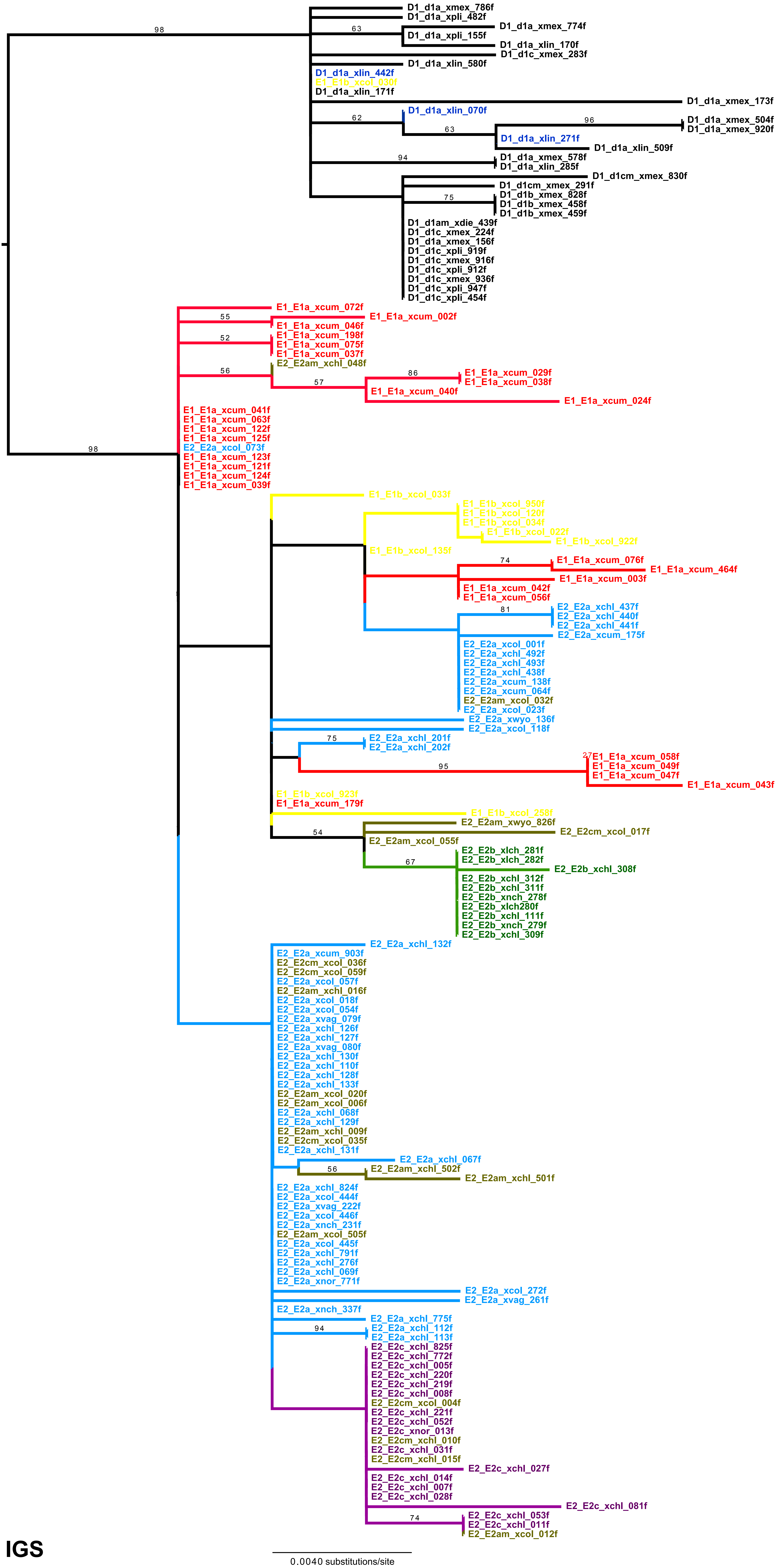
D1_d1a_xmex_786f
D1_d1a_xpli_482f
D1_d1a_xmex_774f
D1_d1a_xpli_155f
D1_d1a_xlin_170f
D1_d1c_xmex_283f
D1_d1a_xlin_580f
D1_d1a_xlin_442f
E1_E1b_xcol_030f
D1_d1a_xlin_171f
D1_d1a_xmex_173f
D1_d1a_xlin_070f
D1_d1a_xmex_504f
D1_d1a_xmex_920f
D1_d1a_xlin_271f
D1_d1a_xlin_509f
D1_d1a_xmex_578f
D1_d1a_xlin_285f
D1_d1cm_xmex_830f
D1_d1cm_xmex_291f
D1_d1b_xmex_828f
D1_d1b_xmex_458f
D1_d1b_xmex_459f
D1_d1am_xdie_439f
D1_d1c_xmex_224f
D1_d1a_xmex_156f
D1_d1c_xpli_919f
D1_d1c_xmex_916f
D1_d1c_xpli_912f
D1_d1c_xmex_936f
D1_d1c_xpli_947f
D1_d1c_xpli_454f
E1_E1a_xcum_072f
E1_E1a_xcum_002f
E1_E1a_xcum_046f
E1_E1a_xcum_198f
E1_E1a_xcum_075f
E1_E1a_xcum_037f
E2_E2am_xchl_048f
E1_E1a_xcum_029f
E1_E1a_xcum_038f
E1_E1a_xcum_040f
E1_E1a_xcum_024f
E1_E1a_xcum_041f
E1_E1a_xcum_063f
E1_E1a_xcum_122f
E1_E1a_xcum_125f
E2_E2a_xcol_073f
E1_E1a_xcum_123f
E1_E1a_xcum_121f
E1_E1a_xcum_124f
E1_E1a_xcum_039f
E1_E1b_xcol_033f
E1_E1b_xcol_950f
E1_E1b_xcol_120f
E1_E1b_xcol_034f
E1_E1b_xcol_022f
E1_E1b_xcol_922f
E1_E1b_xcol_135f
E1_E1a_xcum_076f
E1_E1a_xcum_464f
E1_E1a_xcum_003f
E1_E1a_xcum_042f
E1_E1a_xcum_056f
E2_E2a_xchl_437f
E2_E2a_xchl_440f
E2_E2a_xchl_441f
E2_E2a_xcum_175f
E2_E2a_xcol_001f
E2_E2a_xchl_492f
E2_E2a_xchl_493f
E2_E2a_xchl_438f
E2_E2a_xcum_138f
E2_E2a_xcum_064f
E2_E2am_xcol_032f
E2_E2a_xcol_023f
E2_E2a_xwyo_136f
E2_E2a_xcol_118f
E2_E2a_xchl_201f
E2_E2a_xchl_202f
E1_E1a_xcum_058f
E1_E1a_xcum_049f
E1_E1a_xcum_047f
E1_E1a_xcum_043f
E1_E1b_xcol_923f
E1_E1a_xcum_179f
E1_E1b_xcol_258f
E2_E2am_xwyo_826f
E2_E2cm_xcol_017f
E2_E2am_xcol_055f
E2_E2b_xlch_281f
E2_E2b_xlch_282f
E2_E2b_xchl_308f
E2_E2b_xchl_312f
E2_E2b_xchl_311f
E2_E2b_xnch_278f
E2_E2b_xlch280f
E2_E2b_xchl_111f
E2_E2b_xnch_279f
E2_E2b_xchl_309f
E2_E2a_xchl_132f
E2_E2a_xcum_903f
E2_E2cm_xcol_036f
E2_E2cm_xcol_059f
E2_E2a_xcol_057f
E2_E2am_xchl_016f
E2_E2a_xcol_018f
E2_E2a_xcol_054f
E2_E2a_xvag_079f
E2_E2a_xchl_126f
E2_E2a_xchl_127f
E2_E2a_xvag_080f
E2_E2a_xchl_130f
E2_E2a_xchl_110f
E2_E2a_xchl_128f
E2_E2a_xchl_133f
E2_E2am_xcol_020f
E2_E2am_xcol_006f
E2_E2a_xchl_068f
E2_E2a_xchl_129f
E2_E2am_xchl_009f
E2_E2cm_xcol_035f
E2_E2a_xchl_131f
E2_E2a_xchl_067f
E2_E2am_xchl_502f
E2_E2am_xchl_501f
E2_E2a_xchl_824f
E2_E2a_xcol_444f
E2_E2a_xvag_222f
E2_E2a_xcol_446f
E2_E2a_xnch_231f
E2_E2am_xcol_505f
E2_E2a_xcol_445f
E2_E2a_xchl_791f
E2_E2a_xchl_276f
E2_E2a_xchl_069f
E2_E2a_xnor_771f
E2_E2a_xcol_272f
E2_E2a_xvag_261f
E2_E2a_xnch_337f
E2_E2a_xchl_775f
E2_E2a_xchl_112f
E2_E2a_xchl_113f
E2_E2c_xchl_825f
E2_E2c_xchl_772f
E2_E2c_xchl_005f
E2_E2c_xchl_220f
E2_E2c_xchl_219f
E2_E2c_xchl_008f
E2_E2cm_xcol_004f
E2_E2c_xchl_221f
E2_E2c_xchl_052f
E2_E2c_xnor_013f
E2_E2cm_xchl_010f
E2_E2c_xchl_031f
E2_E2cm_xchl_015f
E2_E2c_xchl_027f
E2_E2c_xchl_014f
E2_E2c_xchl_007f
E2_E2c_xchl_028f
E2_E2c_xchl_081f
E2_E2c_xchl_053f
E2_E2c_xchl_011f
E2_E2am_xcol_012f
IGS
0.0040 substitutions/site

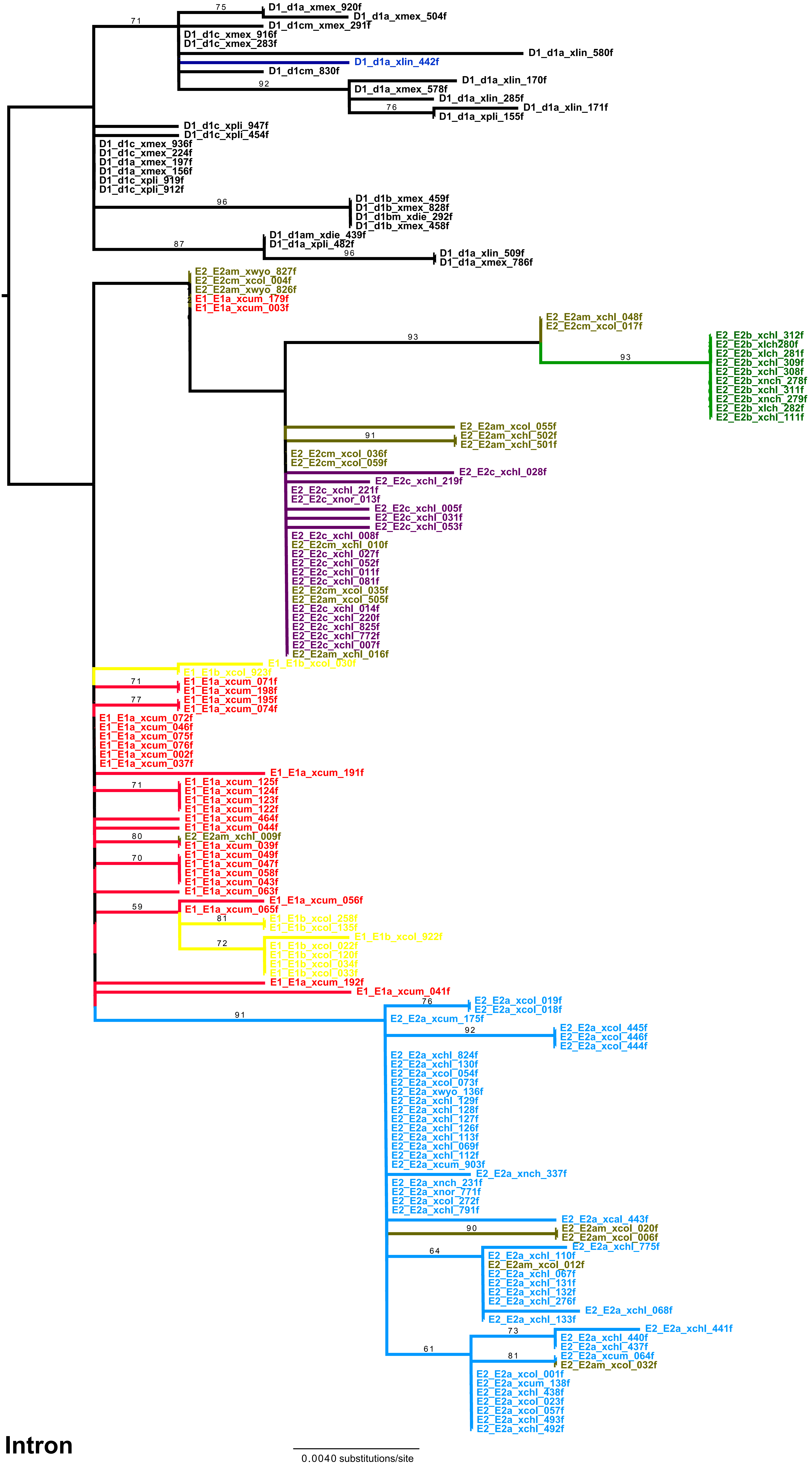
75
71
D1_d1a_xmex_920f
D1_d1a_xmex_504f
D1_d1cm_xmex_291f
D1_d1c_xmex_916f
D1_d1c_xmex_283f
D1_d1a_xlin_580f
D1_d1a_xlin_442f
D1_d1cm_830f
92
D1_d1a_xlin_170f
D1_d1a_xmex_578f
D1_d1a_xlin_285f
76
D1_d1a_xlin_171f
D1_d1a_xpli_155f
D1_d1c_xpli_947f
D1_d1c_xpli_454f
D1_d1c_xmex_936f
D1_d1c_xmex_224f
D1_d1a_xmex_197f
D1_d1a_xmex_156f
D1_d1c_xpli_919f
D1_d1c_xpli_912f
96
D1_d1b_xmex_459f
D1_d1b_xmex_828f
D1_d1bm_xdie_292f
D1_d1b_xmex_458f
87
D1_d1am_xdie_439f
D1_d1a_xpli_482f
96
D1_d1a_xlin_509f
D1_d1a_xmex_786f
E2_E2am_xwyo_827f
E2_E2cm_xcol_004f
E2_E2am_xwyo_826f
E1_E1a_xcum_179f
E1_E1a_xcum_003f
E2_E2am_xchl_048f
E2_E2cm_xcol_017f
93
93
E2_E2b_xchl_312f
E2_E2b_xlch280f
E2_E2b_xlch_281f
E2_E2b_xchl_309f
E2_E2b_xchl_308f
E2_E2b_xnch_278f
E2_E2b_xchl_311f
E2_E2b_xnch_279f
E2_E2b_xlch_282f
E2_E2b_xchl_111f
E2_E2am_xcol_055f
91
E2_E2am_xchl_502f
E2_E2am_xchl_501f
E2_E2cm_xcol_036f
E2_E2cm_xcol_059f
E2_E2c_xchl_028f
E2_E2c_xchl_219f
E2_E2c_xchl_221f
E2_E2c_xnor_013f
E2_E2c_xchl_005f
E2_E2c_xchl_031f
E2_E2c_xchl_053f
E2_E2c_xchl_008f
E2_E2cm_xchl_010f
E2_E2c_xchl_027f
E2_E2c_xchl_052f
E2_E2c_xchl_011f
E2_E2c_xchl_081f
E2_E2cm_xcol_035f
E2_E2am_xcol_505f
E2_E2c_xchl_014f
E2_E2c_xchl_220f
E2_E2c_xchl_825f
E2_E2c_xchl_772f
E2_E2c_xchl_007f
E2_E2am_xchl_016f
E1_E1b_xcol_030f
E1_E1b_xcol_923f
71
E1_E1a_xcum_071f
E1_E1a_xcum_198f
77
E1_E1a_xcum_195f
E1_E1a_xcum_074f
E1_E1a_xcum_072f
E1_E1a_xcum_046f
E1_E1a_xcum_075f
E1_E1a_xcum_076f
E1_E1a_xcum_002f
E1_E1a_xcum_037f
E1_E1a_xcum_191f
71
E1_E1a_xcum_125f
E1_E1a_xcum_124f
E1_E1a_xcum_123f
E1_E1a_xcum_122f
E1_E1a_xcum_464f
E1_E1a_xcum_044f
80
E2_E2am_xchl_009f
E1_E1a_xcum_039f
70
E1_E1a_xcum_049f
E1_E1a_xcum_047f
E1_E1a_xcum_058f
E1_E1a_xcum_043f
E1_E1a_xcum_063f
E1_E1a_xcum_056f
59
E1_E1a_xcum_065f
81
E1_E1b_xcol_258f
E1_E1b_xcol_135f
E1_E1b_xcol_922f
72
E1_E1b_xcol_022f
E1_E1b_xcol_120f
E1_E1b_xcol_034f
E1_E1b_xcol_033f
E1_E1a_xcum_192f
E1_E1a_xcum_041f
76
E2_E2a_xcol_019f
E2_E2a_xcol_018f
91
E2_E2a_xcum_175f
92
E2_E2a_xcol_445f
E2_E2a_xcol_446f
E2_E2a_xcol_444f
E2_E2a_xchl_824f
E2_E2a_xchl_130f
E2_E2a_xcol_054f
E2_E2a_xcol_073f
E2_E2a_xwyo_136f
E2_E2a_xchl_129f
E2_E2a_xchl_128f
E2_E2a_xchl_127f
E2_E2a_xchl_126f
E2_E2a_xchl_113f
E2_E2a_xchl_069f
E2_E2a_xchl_112f
E2_E2a_xcum_903f
E2_E2a_xnch_337f
E2_E2a_xnch_231f
E2_E2a_xnor_771f
E2_E2a_xcol_272f
E2_E2a_xchl_791f
E2_E2a_xcal_443f
90
E2_E2am_xcol_020f
E2_E2am_xcol_006f
E2_E2a_xchl_775f
64
E2_E2a_xchl_110f
E2_E2am_xcol_012f
E2_E2a_xchl_067f
E2_E2a_xchl_131f
E2_E2a_xchl_132f
E2_E2a_xchl_276f
E2_E2a_xchl_068f
E2_E2a_xchl_133f
73
E2_E2a_xchl_441f
E2_E2a_xchl_440f
61
E2_E2a_xchl_437f
81
E2_E2a_xcum_064f
E2_E2am_xcol_032f
E2_E2a_xcol_001f
E2_E2a_xcum_138f
E2_E2a_xchl_438f
E2_E2a_xcol_023f
E2_E2a_xcol_057f
E2_E2a_xchl_493f
E2_E2a_xchl_492f
Intron
0.0040 substitutions/site

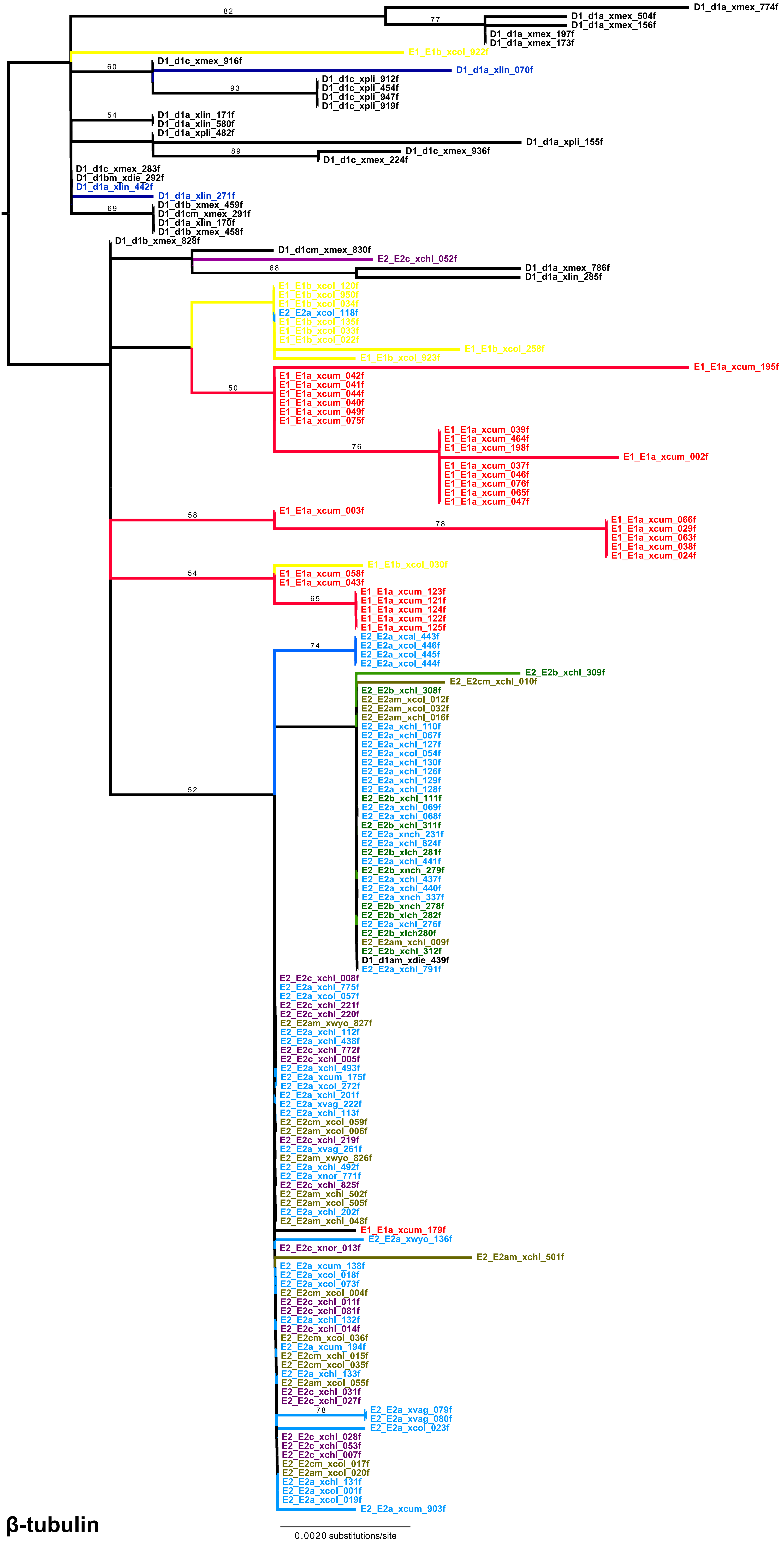

82
D1_d1a_xmex_774f
77
D1_d1a_xmex_504f
D1_d1a_xmex_156f
D1_d1a_xmex_197f
D1_d1a_xmex_173f
E1_E1b_xcol_922f
D1_d1c_xmex_916f
60
D1_d1a_xlin_070f
D1_d1c_xpli_912f
D1_d1c_xpli_454f
93
D1_d1c_xpli_947f
D1_d1c_xpli_919f
54
D1_d1a_xlin_171f
D1_d1a_xlin_580f
D1_d1a_xpli_482f
D1_d1a_xpli_155f
89
D1_d1c_xmex_936f
D1_d1c_xmex_224f
D1_d1c_xmex_283f
D1_d1bm_xdie_292f
D1_d1a_xlin_442f
D1_d1a_xlin_271f
69
D1_d1b_xmex_459f
D1_d1cm_xmex_291f
D1_d1a_xlin_170f
D1_d1b_xmex_458f
D1_d1b_xmex_828f
D1_d1cm_xmex_830f
E2_E2c_xchl_052f
68
D1_d1a_xmex_786f
D1_d1a_xlin_285f
E1_E1b_xcol_120f
E1_E1b_xcol_950f
E1_E1b_xcol_034f
E2_E2a_xcol_118f
E1_E1b_xcol_135f
E1_E1b_xcol_033f
E1_E1b_xcol_022f
E1_E1b_xcol_258f
E1_E1b_xcol_923f
E1_E1a_xcum_195f
E1_E1a_xcum_042f
E1_E1a_xcum_041f
50
E1_E1a_xcum_044f
E1_E1a_xcum_040f
E1_E1a_xcum_049f
E1_E1a_xcum_075f
E1_E1a_xcum_039f
E1_E1a_xcum_464f
76
E1_E1a_xcum_198f
E1_E1a_xcum_002f
E1_E1a_xcum_037f
E1_E1a_xcum_046f
E1_E1a_xcum_076f
E1_E1a_xcum_065f
E1_E1a_xcum_047f
58
E1_E1a_xcum_003f
E1_E1a_xcum_066f
78
E1_E1a_xcum_029f
E1_E1a_xcum_063f
E1_E1a_xcum_038f
E1_E1a_xcum_024f
E1_E1b_xcol_030f
54
E1_E1a_xcum_058f
E1_E1a_xcum_043f
E1_E1a_xcum_123f
65
E1_E1a_xcum_121f
E1_E1a_xcum_124f
E1_E1a_xcum_122f
E1_E1a_xcum_125f
E2_E2a_xcal_443f
74
E2_E2a_xcol_446f
E2_E2a_xcol_445f
E2_E2a_xcol_444f
E2_E2b_xchl_309f
E2_E2cm_xchl_010f
E2_E2b_xchl_308f
E2_E2am_xcol_012f
E2_E2am_xcol_032f
E2_E2am_xchl_016f
E2_E2a_xchl_110f
E2_E2a_xchl_067f
E2_E2a_xchl_127f
E2_E2a_xcol_054f
E2_E2a_xchl_130f
E2_E2a_xchl_126f
E2_E2a_xchl_129f
E2_E2a_xchl_128f
52
E2_E2b_xchl_111f
E2_E2a_xchl_069f
E2_E2a_xchl_068f
E2_E2b_xchl_311f
E2_E2a_xnch_231f
E2_E2a_xchl_824f
E2_E2b_xlch_281f
E2_E2a_xchl_441f
E2_E2b_xnch_279f
E2_E2a_xchl_437f
E2_E2a_xchl_440f
E2_E2a_xnch_337f
E2_E2b_xnch_278f
E2_E2b_xlch_282f
E2_E2a_xchl_276f
E2_E2b_xlch280f
E2_E2am_xchl_009f
E2_E2b_xchl_312f
D1_d1am_xdie_439f
E2_E2a_xchl_791f
E2_E2c_xchl_008f
E2_E2a_xchl_775f
E2_E2a_xcol_057f
E2_E2c_xchl_221f
E2_E2c_xchl_220f
E2_E2am_xwyo_827f
E2_E2a_xchl_112f
E2_E2a_xchl_438f
E2_E2c_xchl_772f
E2_E2c_xchl_005f
E2_E2a_xchl_493f
E2_E2a_xcum_175f
E2_E2a_xcol_272f
E2_E2a_xchl_201f
E2_E2a_xvag_222f
E2_E2a_xchl_113f
E2_E2cm_xcol_059f
E2_E2am_xcol_006f
E2_E2c_xchl_219f
E2_E2a_xvag_261f
E2_E2am_xwyo_826f
E2_E2a_xchl_492f
E2_E2a_xnor_771f
E2_E2c_xchl_825f
E2_E2am_xchl_502f
E2_E2am_xcol_505f
E2_E2a_xchl_202f
E2_E2am_xchl_048f
E1_E1a_xcum_179f
E2_E2a_xwyo_136f
E2_E2c_xnor_013f
E2_E2am_xchl_501f
E2_E2a_xcum_138f
E2_E2a_xcol_018f
E2_E2a_xcol_073f
E2_E2cm_xcol_004f
E2_E2c_xchl_011f
E2_E2c_xchl_081f
E2_E2a_xchl_132f
E2_E2c_xchl_014f
E2_E2cm_xcol_036f
E2_E2a_xcum_194f
E2_E2cm_xchl_015f
E2_E2cm_xcol_035f
E2_E2a_xchl_133f
E2_E2am_xcol_055f
E2_E2c_xchl_031f
E2_E2c_xchl_027f
78
E2_E2a_xvag_079f
E2_E2a_xvag_080f
E2_E2a_xcol_023f
E2_E2c_xchl_028f
E2_E2c_xchl_053f
E2_E2c_xchl_007f
E2_E2cm_xcol_017f
E2_E2am_xcol_020f
E2_E2a_xchl_131f
E2_E2a_xcol_001f
E2_E2a_xcol_019f
E2_E2a_xcum_903f
0.0020 substitutions/site

β-tubulin

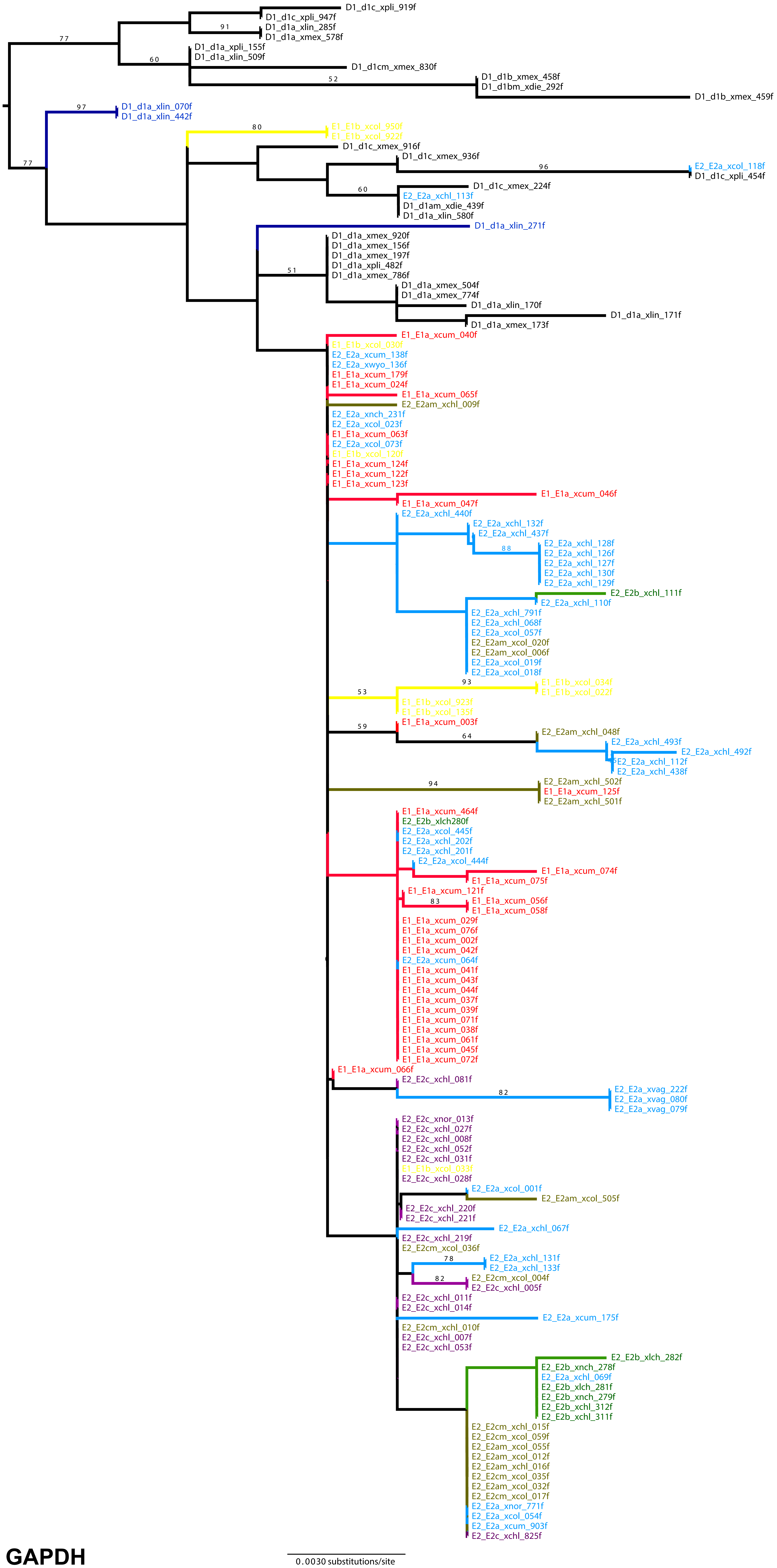
D1_d1c_xpli_919f
D1_d1c_xpli_947f
91
D1_d1a_xlin_285f
D1_d1a_xmex_578f
77
D1_d1a_xpli_155f
60
D1_d1a_xlin_509f
D1_d1cm_xmex_830f
D1_d1b_xmex_458f
52
D1_d1bm_xdie_292f
D1_d1b_xmex_459f
97
D1_d1a_xlin_070f
D1_d1a_xlin_442f
80
E1_E1b_xcol_950f
E1_E1b_xcol_922f
D1_d1c_xmex_916f
77
D1_d1c_xmex_936f
96
E2_E2a_xcol_118f
D1_d1c_xpli_454f
D1_d1c_xmex_224f
60
E2_E2a_xchl_113f
D1_d1am_xdie_439f
D1_d1a_xlin_580f
D1_d1a_xlin_271f
D1_d1a_xmex_920f
D1_d1a_xmex_156f
D1_d1a_xmex_197f
51
D1_d1a_xpli_482f
D1_d1a_xmex_786f
D1_d1a_xmex_504f
D1_d1a_xmex_774f
D1_d1a_xlin_170f
D1_d1a_xlin_171f
D1_d1a_xmex_173f
E1_E1a_xcum_040f
E1_E1b_xcol_030f
E2_E2a_xcum_138f
E2_E2a_xwyo_136f
E1_E1a_xcum_179f
E1_E1a_xcum_024f
E1_E1a_xcum_065f
E2_E2am_xchl_009f
E2_E2a_xnch_231f
E2_E2a_xcol_023f
E1_E1a_xcum_063f
E2_E2a_xcol_073f
E1_E1b_xcol_120f
E1_E1a_xcum_124f
E1_E1a_xcum_122f
E1_E1a_xcum_123f
E1_E1a_xcum_046f
E1_E1a_xcum_047f
E2_E2a_xchl_440f
E2_E2a_xchl_132f
E2_E2a_xchl_437f
E2_E2a_xchl_128f
88
E2_E2a_xchl_126f
E2_E2a_xchl_127f
E2_E2a_xchl_130f
E2_E2a_xchl_129f
E2_E2b_xchl_111f
E2_E2a_xchl_110f
E2_E2a_xchl_791f
E2_E2a_xchl_068f
E2_E2a_xcol_057f
E2_E2am_xcol_020f
E2_E2am_xcol_006f
E2_E2a_xcol_019f
E2_E2a_xcol_018f
93
E1_E1b_xcol_034f
53
E1_E1b_xcol_022f
E1_E1b_xcol_923f
E1_E1b_xcol_135f
59
E1_E1a_xcum_003f
E2_E2am_xchl_048f
64
E2_E2a_xchl_493f
E2_E2a_xchl_492f
E2_E2a_xchl_112f
E2_E2a_xchl_438f
94
E2_E2am_xchl_502f
E1_E1a_xcum_125f
E2_E2am_xchl_501f
E1_E1a_xcum_464f
E2_E2b_xlch280f
E2_E2a_xcol_445f
E2_E2a_xchl_202f
E2_E2a_xchl_201f
E2_E2a_xcol_444f
E1_E1a_xcum_074f
E1_E1a_xcum_075f
E1_E1a_xcum_121f
83
E1_E1a_xcum_056f
E1_E1a_xcum_058f
E1_E1a_xcum_029f
E1_E1a_xcum_076f
E1_E1a_xcum_002f
E1_E1a_xcum_042f
E2_E2a_xcum_064f
E1_E1a_xcum_041f
E1_E1a_xcum_043f
E1_E1a_xcum_044f
E1_E1a_xcum_037f
E1_E1a_xcum_039f
E1_E1a_xcum_071f
E1_E1a_xcum_038f
E1_E1a_xcum_061f
E1_E1a_xcum_045f
E1_E1a_xcum_072f
E1_E1a_xcum_066f
E2_E2c_xchl_081f
82
E2_E2a_xvag_222f
E2_E2a_xvag_080f
E2_E2a_xvag_079f
E2_E2c_xnor_013f
E2_E2c_xchl_027f
E2_E2c_xchl_008f
E2_E2c_xchl_052f
E2_E2c_xchl_031f
E1_E1b_xcol_033f
E2_E2c_xchl_028f
E2_E2a_xcol_001f
E2_E2am_xcol_505f
E2_E2c_xchl_220f
E2_E2c_xchl_221f
E2_E2a_xchl_067f
E2_E2c_xchl_219f
E2_E2cm_xcol_036f
78
E2_E2a_xchl_131f
E2_E2a_xchl_133f
82
E2_E2cm_xcol_004f
E2_E2c_xchl_005f
E2_E2c_xchl_011f
E2_E2c_xchl_014f
E2_E2a_xcum_175f
E2_E2cm_xchl_010f
E2_E2c_xchl_007f
E2_E2c_xchl_053f
E2_E2b_xlch_282f
E2_E2b_xnch_278f
E2_E2a_xchl_069f
E2_E2b_xlch_281f
E2_E2b_xnch_279f
E2_E2b_xchl_312f
E2_E2b_xchl_311f
E2_E2cm_xchl_015f
E2_E2cm_xcol_059f
E2_E2am_xcol_055f
E2_E2am_xcol_012f
E2_E2am_xchl_016f
E2_E2cm_xcol_035f
E2_E2am_xcol_032f
E2_E2cm_xcol_017f
E2_E2a_xnor_771f
E2_E2a_xcol_054f
E2_E2a_xcum_903f
E2_E2c_xchl_825f
GAPDH
0.0030 substitutions/site

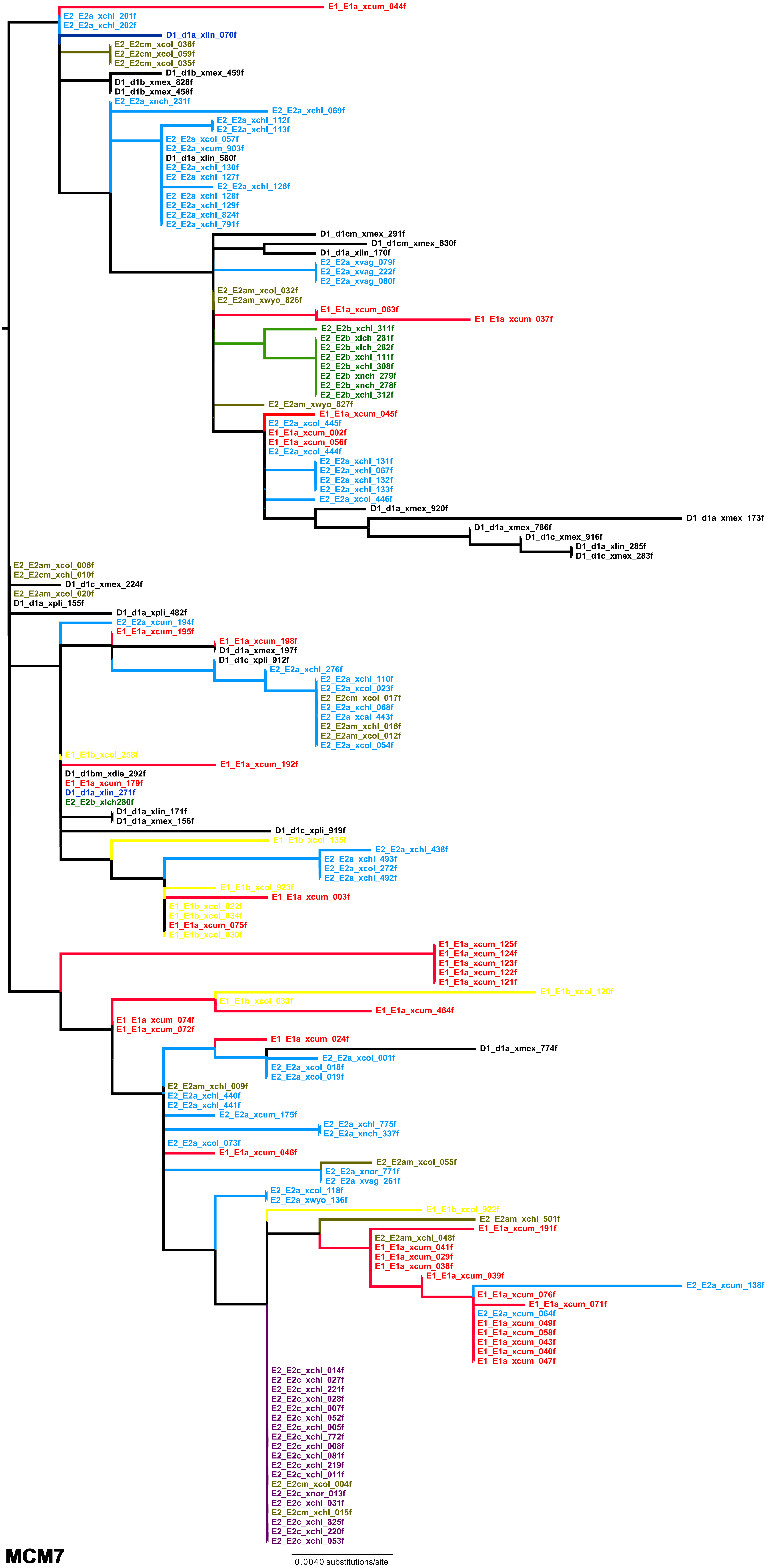
E1_E1a_xcum_044f
E2_E2a_xchl_201f
E2_E2a_xchl_202f
D1_d1a_xlin_070f
E2_E2cm_xcol_036f
E2_E2cm_xcol_059f
E2_E2cm_xcol_035f
D1_d1b_xmex_459f
D1_d1b_xmex_828f
D1_d1b_xmex_458f
E2_E2a_xnch_231f
E2_E2a_xchl_069f
E2_E2a_xchl_112f
E2_E2a_xchl_113f
E2_E2a_xcol_057f
E2_E2a_xcum_903f
D1_d1a_xlin_580f
E2_E2a_xchl_130f
E2_E2a_xchl_127f
E2_E2a_xchl_126f
E2_E2a_xchl_128f
E2_E2a_xchl_129f
E2_E2a_xchl_824f
E2_E2a_xchl_791f
D1_d1cm_xmex_291f
D1_d1cm_xmex_830f
D1_d1a_xlin_170f
E2_E2a_xvag_079f
E2_E2a_xvag_222f
E2_E2a_xvag_080f
E2_E2am_xcol_032f
E2_E2am_xwyo_826f
E1_E1a_xcum_063f
E1_E1a_xcum_037f
E2_E2b_xchl_311f
E2_E2b_xlch_281f
E2_E2b_xlch_282f
E2_E2b_xchl_111f
E2_E2b_xchl_308f
E2_E2b_xnch_279f
E2_E2b_xnch_278f
E2_E2b_xchl_312f
E2_E2am_xwyo_827f
E1_E1a_xcum_045f
E2_E2a_xcol_445f
E1_E1a_xcum_002f
E1_E1a_xcum_056f
E2_E2a_xcol_444f
E2_E2a_xchl_131f
E2_E2a_xchl_067f
E2_E2a_xchl_132f
E2_E2a_xchl_133f
E2_E2a_xcol_446f
D1_d1a_xmex_920f
D1_d1a_xmex_173f
D1_d1a_xmex_786f
D1_d1c_xmex_916f
D1_d1a_xlin_285f
D1_d1c_xmex_283f
E2_E2am_xcol_006f
E2_E2cm_xchl_010f
D1_d1c_xmex_224f
E2_E2am_xcol_020f
D1_d1a_xpli_155f
D1_d1a_xpli_482f
E2_E2a_xcum_194f
E1_E1a_xcum_195f
E1_E1a_xcum_198f
D1_d1a_xmex_197f
D1_d1c_xpli_912f
E2_E2a_xchl_276f
E2_E2a_xchl_110f
E2_E2a_xcol_023f
E2_E2cm_xcol_017f
E2_E2a_xchl_068f
E2_E2a_xcal_443f
E2_E2am_xchl_016f
E2_E2am_xcol_012f
E2_E2a_xcol_054f
E1_E1b_xcol_258f
E1_E1a_xcum_192f
D1_d1bm_xdie_292f
E1_E1a_xcum_179f
D1_d1a_xlin_271f
E2_E2b_xlch280f
D1_d1a_xlin_171f
D1_d1a_xmex_156f
D1_d1c_xpli_919f
E1_E1b_xcol_135f
E2_E2a_xchl_438f
E2_E2a_xchl_493f
E2_E2a_xcol_272f
E2_E2a_xchl_492f
E1_E1b_xcol_923f
E1_E1a_xcum_003f
E1_E1b_xcol_022f
E1_E1b_xcol_034f
E1_E1a_xcum_075f
E1_E1b_xcol_030f
E1_E1a_xcum_125f
E1_E1a_xcum_124f
E1_E1a_xcum_123f
E1_E1a_xcum_122f
E1_E1a_xcum_121f
E1_E1b_xcol_120f
E1_E1b_xcol_033f
E1_E1a_xcum_464f
E1_E1a_xcum_074f
E1_E1a_xcum_072f
E1_E1a_xcum_024f
D1_d1a_xmex_774f
E2_E2a_xcol_001f
E2_E2a_xcol_018f
E2_E2a_xcol_019f
E2_E2am_xchl_009f
E2_E2a_xchl_440f
E2_E2a_xchl_441f
E2_E2a_xcum_175f
E2_E2a_xchl_775f
E2_E2a_xnch_337f
E2_E2a_xcol_073f
E1_E1a_xcum_046f
E2_E2am_xcol_055f
E2_E2a_xnor_771f
E2_E2a_xvag_261f
E2_E2a_xcol_118f
E2_E2a_xwyo_136f
E1_E1b_xcol_922f
E2_E2am_xchl_501f
E1_E1a_xcum_191f
E2_E2am_xchl_048f
E1_E1a_xcum_041f
E1_E1a_xcum_029f
E1_E1a_xcum_038f
E1_E1a_xcum_039f
E2_E2a_xcum_138f
E1_E1a_xcum_076f
E1_E1a_xcum_071f
E2_E2a_xcum_064f
E1_E1a_xcum_049f
E1_E1a_xcum_058f
E1_E1a_xcum_043f
E1_E1a_xcum_040f
E1_E1a_xcum_047f
E2_E2c_xchl_014f
E2_E2c_xchl_027f
E2_E2c_xchl_221f
E2_E2c_xchl_028f
E2_E2c_xchl_007f
E2_E2c_xchl_052f
E2_E2c_xchl_005f
E2_E2c_xchl_772f
E2_E2c_xchl_008f
E2_E2c_xchl_081f
E2_E2c_xchl_219f
E2_E2c_xchl_011f
E2_E2cm_xcol_004f
E2_E2c_xnor_013f
E2_E2c_xchl_031f
E2_E2cm_xchl_015f
E2_E2c_xchl_825f
E2_E2c_xchl_220f
E2_E2c_xchl_053f
MCM7
0.0040 substitutions/site

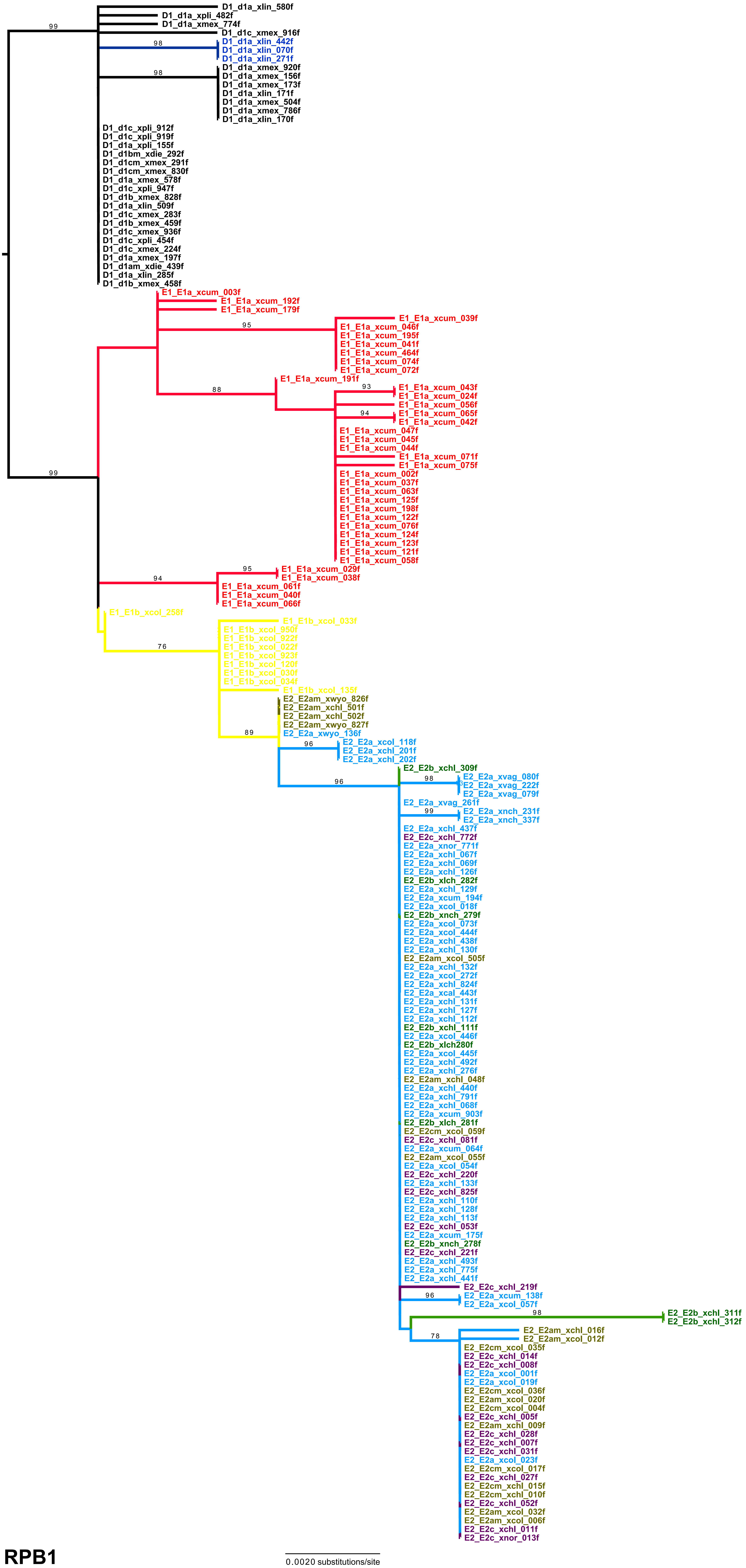
D1_d1a_xlin_580f
D1_d1a_xpli_482f
D1_d1a_xmex_774f
D1_d1c_xmex_916f
D1_d1a_xlin_442f
D1_d1a_xlin_070f
D1_d1a_xlin_271f
D1_d1a_xmex_920f
D1_d1a_xmex_156f
D1_d1a_xmex_173f
D1_d1a_xlin_171f
D1_d1a_xmex_504f
D1_d1a_xmex_786f
D1_d1a_xlin_170f
D1_d1c_xpli_912f
D1_d1c_xpli_919f
D1_d1a_xpli_155f
D1_d1bm_xdie_292f
D1_d1cm_xmex_291f
D1_d1cm_xmex_830f
D1_d1a_xmex_578f
D1_d1c_xpli_947f
D1_d1b_xmex_828f
D1_d1a_xlin_509f
D1_d1c_xmex_283f
D1_d1b_xmex_459f
D1_d1c_xmex_936f
D1_d1c_xpli_454f
D1_d1c_xmex_224f
D1_d1a_xmex_197f
D1_d1am_xdie_439f
D1_d1a_xlin_285f
D1_d1b_xmex_458f
E1_E1a_xcum_003f
E1_E1a_xcum_192f
E1_E1a_xcum_179f
E1_E1a_xcum_039f
E1_E1a_xcum_046f
E1_E1a_xcum_195f
E1_E1a_xcum_041f
E1_E1a_xcum_464f
E1_E1a_xcum_074f
E1_E1a_xcum_072f
E1_E1a_xcum_191f
E1_E1a_xcum_043f
E1_E1a_xcum_024f
E1_E1a_xcum_056f
E1_E1a_xcum_065f
E1_E1a_xcum_042f
E1_E1a_xcum_047f
E1_E1a_xcum_045f
E1_E1a_xcum_044f
E1_E1a_xcum_071f
E1_E1a_xcum_075f
E1_E1a_xcum_002f
E1_E1a_xcum_037f
E1_E1a_xcum_063f
E1_E1a_xcum_125f
E1_E1a_xcum_198f
E1_E1a_xcum_122f
E1_E1a_xcum_076f
E1_E1a_xcum_124f
E1_E1a_xcum_123f
E1_E1a_xcum_121f
E1_E1a_xcum_058f
E1_E1a_xcum_029f
E1_E1a_xcum_038f
E1_E1a_xcum_061f
E1_E1a_xcum_040f
E1_E1a_xcum_066f
E1_E1b_xcol_258f
E1_E1b_xcol_033f
E1_E1b_xcol_950f
E1_E1b_xcol_922f
E1_E1b_xcol_022f
E1_E1b_xcol_923f
E1_E1b_xcol_120f
E1_E1b_xcol_030f
E1_E1b_xcol_034f
E1_E1b_xcol_135f
E2_E2am_xwyo_826f
E2_E2am_xchl_501f
E2_E2am_xchl_502f
E2_E2am_xwyo_827f
E2_E2a_xwyo_136f
E2_E2a_xcol_118f
E2_E2a_xchl_201f
E2_E2a_xchl_202f
E2_E2b_xchl_309f
E2_E2a_xvag_080f
E2_E2a_xvag_222f
E2_E2a_xvag_079f
E2_E2a_xvag_261f
E2_E2a_xnch_231f
E2_E2a_xnch_337f
E2_E2a_xchl_437f
E2_E2c_xchl_772f
E2_E2a_xnor_771f
E2_E2a_xchl_067f
E2_E2a_xchl_069f
E2_E2a_xchl_126f
E2_E2b_xlch_282f
E2_E2a_xchl_129f
E2_E2a_xcum_194f
E2_E2a_xcol_018f
E2_E2b_xnch_279f
E2_E2a_xcol_073f
E2_E2a_xcol_444f
E2_E2a_xchl_438f
E2_E2a_xchl_130f
E2_E2am_xcol_505f
E2_E2a_xchl_132f
E2_E2a_xcol_272f
E2_E2a_xchl_824f
E2_E2a_xcal_443f
E2_E2a_xchl_131f
E2_E2a_xchl_127f
E2_E2a_xchl_112f
E2_E2b_xchl_111f
E2_E2a_xcol_446f
E2_E2b_xlch280f
E2_E2a_xcol_445f
E2_E2a_xchl_492f
E2_E2a_xchl_276f
E2_E2am_xchl_048f
E2_E2a_xchl_440f
E2_E2a_xchl_791f
E2_E2a_xchl_068f
E2_E2a_xcum_903f
E2_E2b_xlch_281f
E2_E2cm_xcol_059f
E2_E2c_xchl_081f
E2_E2a_xcum_064f
E2_E2am_xcol_055f
E2_E2a_xcol_054f
E2_E2c_xchl_220f
E2_E2a_xchl_133f
E2_E2c_xchl_825f
E2_E2a_xchl_110f
E2_E2a_xchl_128f
E2_E2a_xchl_113f
E2_E2c_xchl_053f
E2_E2a_xcum_175f
E2_E2b_xnch_278f
E2_E2c_xchl_221f
E2_E2a_xchl_493f
E2_E2a_xchl_775f
E2_E2a_xchl_441f
E2_E2c_xchl_219f
E2_E2a_xcum_138f
E2_E2a_xcol_057f
E2_E2b_xchl_311f
E2_E2b_xchl_312f
E2_E2am_xchl_016f
E2_E2am_xcol_012f
E2_E2cm_xcol_035f
E2_E2c_xchl_014f
E2_E2c_xchl_008f
E2_E2a_xcol_001f
E2_E2a_xcol_019f
E2_E2cm_xcol_036f
E2_E2am_xcol_020f
E2_E2cm_xcol_004f
E2_E2c_xchl_005f
E2_E2am_xchl_009f
E2_E2c_xchl_028f
E2_E2c_xchl_007f
E2_E2c_xchl_031f
E2_E2a_xcol_023f
E2_E2cm_xcol_017f
E2_E2c_xchl_027f
E2_E2cm_xchl_015f
E2_E2cm_xchl_010f
E2_E2c_xchl_052f
E2_E2am_xcol_032f
E2_E2am_xcol_006f
E2_E2c_xchl_011f
E2_E2c_xnor_013f
RPB1
0.0020 substitutions/site

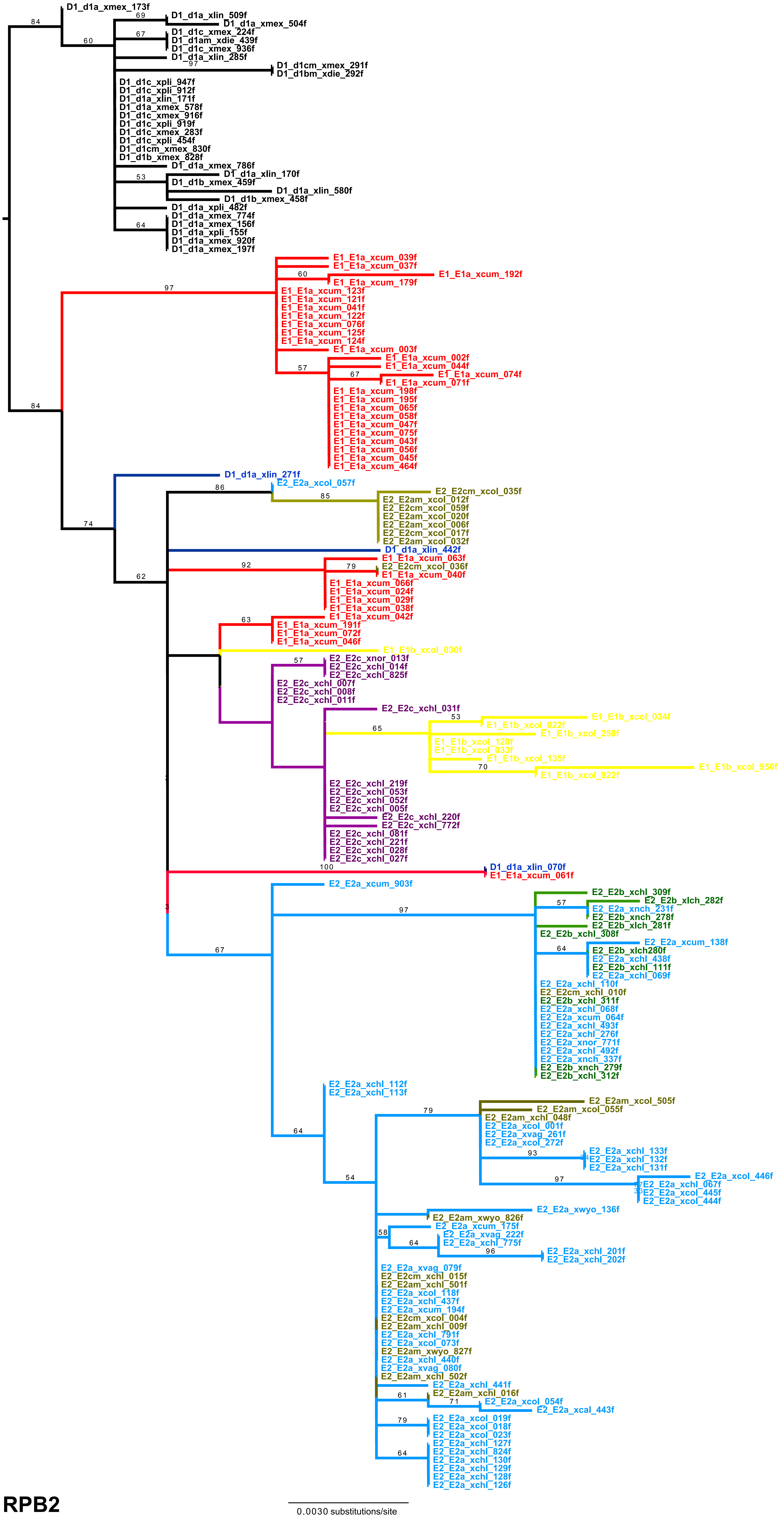
D1_d1a_xmex_173f
D1_d1a_xlin_509f
D1_d1a_xmex_504f
D1_d1c_xmex_224f
D1_d1am_xdie_439f
D1_d1c_xmex_936f
D1_d1a_xlin_285f
D1_d1cm_xmex_291f
D1_d1bm_xdie_292f
D1_d1c_xpli_947f
D1_d1c_xpli_912f
D1_d1a_xlin_171f
D1_d1a_xmex_578f
D1_d1c_xmex_916f
D1_d1c_xpli_919f
D1_d1c_xmex_283f
D1_d1c_xpli_454f
D1_d1cm_xmex_830f
D1_d1b_xmex_828f
D1_d1a_xmex_786f
D1_d1a_xlin_170f
D1_d1b_xmex_459f
D1_d1a_xlin_580f
D1_d1b_xmex_458f
D1_d1a_xpli_482f
D1_d1a_xmex_774f
D1_d1a_xmex_156f
D1_d1a_xpli_155f
D1_d1a_xmex_920f
D1_d1a_xmex_197f
E1_E1a_xcum_039f
E1_E1a_xcum_037f
E1_E1a_xcum_192f
E1_E1a_xcum_179f
E1_E1a_xcum_123f
E1_E1a_xcum_121f
E1_E1a_xcum_041f
E1_E1a_xcum_122f
E1_E1a_xcum_076f
E1_E1a_xcum_125f
E1_E1a_xcum_124f
E1_E1a_xcum_003f
E1_E1a_xcum_002f
E1_E1a_xcum_044f
E1_E1a_xcum_074f
E1_E1a_xcum_071f
E1_E1a_xcum_198f
E1_E1a_xcum_195f
E1_E1a_xcum_065f
E1_E1a_xcum_058f
E1_E1a_xcum_047f
E1_E1a_xcum_075f
E1_E1a_xcum_043f
E1_E1a_xcum_056f
E1_E1a_xcum_045f
E1_E1a_xcum_464f
D1_d1a_xlin_271f
E2_E2a_xcol_057f
E2_E2cm_xcol_035f
E2_E2am_xcol_012f
E2_E2cm_xcol_059f
E2_E2am_xcol_020f
E2_E2am_xcol_006f
E2_E2cm_xcol_017f
E2_E2am_xcol_032f
D1_d1a_xlin_442f
E1_E1a_xcum_063f
E2_E2cm_xcol_036f
E1_E1a_xcum_040f
E1_E1a_xcum_066f
E1_E1a_xcum_024f
E1_E1a_xcum_029f
E1_E1a_xcum_038f
E1_E1a_xcum_042f
E1_E1a_xcum_191f
E1_E1a_xcum_072f
E1_E1a_xcum_046f
E1_E1b_xcol_030f
E2_E2c_xnor_013f
E2_E2c_xchl_014f
E2_E2c_xchl_825f
E2_E2c_xchl_007f
E2_E2c_xchl_008f
E2_E2c_xchl_011f
E2_E2c_xchl_031f
E1_E1b_xcol_034f
E1_E1b_xcol_022f
E1_E1b_xcol_258f
E1_E1b_xcol_120f
E1_E1b_xcol_033f
E1_E1b_xcol_135f
E1_E1b_xcol_950f
E1_E1b_xcol_922f
E2_E2c_xchl_219f
E2_E2c_xchl_053f
E2_E2c_xchl_052f
E2_E2c_xchl_005f
E2_E2c_xchl_220f
E2_E2c_xchl_772f
E2_E2c_xchl_081f
E2_E2c_xchl_221f
E2_E2c_xchl_028f
E2_E2c_xchl_027f
D1_d1a_xlin_070f
E1_E1a_xcum_061f
E2_E2a_xcum_903f
E2_E2b_xchl_309f
E2_E2b_xlch_282f
E2_E2a_xnch_231f
E2_E2b_xnch_278f
E2_E2b_xlch_281f
E2_E2b_xchl_308f
E2_E2a_xcum_138f
E2_E2b_xlch280f
E2_E2a_xchl_438f
E2_E2b_xchl_111f
E2_E2a_xchl_069f
E2_E2a_xchl_110f
E2_E2cm_xchl_010f
E2_E2b_xchl_311f
E2_E2a_xchl_068f
E2_E2a_xcum_064f
E2_E2a_xchl_493f
E2_E2a_xchl_276f
E2_E2a_xnor_771f
E2_E2a_xchl_492f
E2_E2a_xnch_337f
E2_E2b_xnch_279f
E2_E2b_xchl_312f
E2_E2a_xchl_112f
E2_E2a_xchl_113f
E2_E2am_xcol_505f
E2_E2am_xcol_055f
E2_E2am_xchl_048f
E2_E2a_xcol_001f
E2_E2a_xvag_261f
E2_E2a_xcol_272f
E2_E2a_xchl_133f
E2_E2a_xchl_132f
E2_E2a_xchl_131f
E2_E2a_xcol_446f
E2_E2a_xchl_067f
E2_E2a_xcol_445f
E2_E2a_xcol_444f
E2_E2a_xwyo_136f
E2_E2am_xwyo_826f
E2_E2a_xcum_175f
E2_E2a_xvag_222f
E2_E2a_xchl_775f
E2_E2a_xchl_201f
E2_E2a_xchl_202f
E2_E2a_xvag_079f
E2_E2cm_xchl_015f
E2_E2am_xchl_501f
E2_E2a_xcol_118f
E2_E2a_xchl_437f
E2_E2a_xcum_194f
E2_E2cm_xcol_004f
E2_E2am_xchl_009f
E2_E2a_xchl_791f
E2_E2a_xcol_073f
E2_E2am_xwyo_827f
E2_E2a_xchl_440f
E2_E2a_xvag_080f
E2_E2am_xchl_502f
E2_E2a_xchl_441f
E2_E2am_xchl_016f
E2_E2a_xcol_054f
E2_E2a_xcal_443f
E2_E2a_xcol_019f
E2_E2a_xcol_018f
E2_E2a_xcol_023f
E2_E2a_xchl_127f
E2_E2a_xchl_824f
E2_E2a_xchl_130f
E2_E2a_xchl_129f
E2_E2a_xchl_128f
E2_E2a_xchl_126f
0.0030 substitutions/site

RPB2
